# Metagenomic insights reveal the differences in the community composition and functional characteristics of the sea turtle microbiomes based on host species and tissue region

**DOI:** 10.3389/fmicb.2025.1652229

**Published:** 2025-10-03

**Authors:** Lingzhi Dong, Yu Du, Feifei Qiu, Meng Zhang, Xiaoxia Wang, Xiaming Zhu, Yuntao Yao, Jinyi Li, Xiang Ji, Xiong Zhu

**Affiliations:** ^1^Clinical and Central Laboratory of Sanya People’s Hospital, Sanya, China; ^2^Hainan Key Laboratory for Herpetological Research, College of Fisheries and Life Sciences, Hainan Tropical Ocean University, Sanya, China; ^3^Department of Radiology, Sanya People’s Hospital, Sanya, China; ^4^Herpetological Research Center, College of Life and Environmental Sciences, Hangzhou Normal University, Hangzhou, China; ^5^Zhejiang Provincial Key Laboratory for Water Environment and Marine Biological Resources Protection, College of Life and Environmental Sciences, Wenzhou University, Wenzhou, China

**Keywords:** sea turtles, *Chelonia mydas*, *Caretta caretta*, *Eretmochelys imbricata*, metagenomic microbiome

## Abstract

**Introduction and methods:**

Sea turtles have been proposed as health indicators of marine ecosystems for their characteristic of longevity and migratory, but they are facing serious threats due to various factors. The microbial communities within animals play an important role in health and disease. Our study aims to explore a thorough evaluation of the sea turtle microbiome by examining the oral, nasal, and cloacal microbial communities of three species: green turtles, hawksbills, and loggerheads, through metagenomic sequencing.

**Results:**

Utilizing approximately 705.81 GB of metagenomic sequencing data from 63 samples collected from different turtle species and tissue regions, we created a nonredundant sea turtle microbial gene catalog (STMGC) containing 10,733,232 unique genes through the de-redundancy of open reading frames (ORFs). Our findings revealed that the sea turtle microbiomes were primarily composed of *Pseudomonadota* (formerly *Proteobacteria*) and *Bacteroidota* (formerly *Bacteroidetes*). The tissue region was a key factor affecting the variability in the sea turtle microbiome, with green turtles showing notable differences among the three turtle species. *Pseudomonadota* was significantly more abundant in oral samples, while *Bacteroidota* was more prevalent in nasal samples. *Campylobacterota* was identified as significantly more abundant in cloacal samples. Importantly, we discovered 389 genera and 1,445 species of potential pathogens within the sea turtle microbiome, indicating potential pathogenic risks that warrant further investigation alongside culturomics. Additionally, our study highlighted significant functional differences among the three turtles and tissue regions. It is worth noting that among the three sea turtles, antibiotic resistance genes are more prevalent in hawksbills, while virulence genes are more abundant in loggerheads. Moreover, within the three tissue regions, antibiotic resistance genes are higher in oral samples, while virulence genes are more extensive in cloacal samples.

**Conclusion:**

The findings in our study demonstrate that the microbial composition and function in these sea turtles exhibit both species-specific and region-specific variations. The implications of these associations and the underlying mechanisms not only provide valuable insights for future studies on the microbial communities of turtles, but also lay the foundation for further research on the health interrelationships among sea turtles, marine and terrestrial animals, humans and the environment, and for defining “One Health” factors.

## Introduction

Sea turtles, a type of marine reptile, have complex life cycles as a long-lived species. As they grow and migrate across oceans, they experience significant changes in their habitats and diets, which could reflect the long-term effects of environmental changes and pollution (Drane et al., 2021). The characteristic makes them ideal biological indicators for monitoring environmental pollutants, and various biological samples such as blood, fat tissue, and eggs have been used for environmental pollutant monitoring (Fraga et al., 2018; Figgener et al., 2019). In addition, the position of sea turtles in the food chain and their extensive distribution in the marine environment enable them to accumulate and reflect heavy metals, organic pollutants, pathogenic infection, and antibiotic resistance (Kraemer et al., 2019; Al-Bahry et al., 2011; Al-Bahry et al., 2009; Conrad et al., 2023). Their migratory habits mean that they may act as a medium for the transmission of pathogens and resistance between different geographical regions, thereby affecting the extensive marine ecosystem. The detection of opportunistic pathogens and antibiotic resistance in sea turtles provides valuable information for the protection of sea turtles and the safety of marine environments (Ebani, 2023; Conrad et al., 2023; Ahasan et al., 2018; Al-Bahry et al., 2011).

Research increasingly shows that sea turtles are threatened by a variety of factors, such as consuming marine debris, the loss and pollution of nesting and feeding habitats due to urban development, predation on nests and hatchlings by both wildlife and domestic animals, collisions with vessels, traditional hunting and egg harvesting, the effects of climate change on marine and land environments, and entanglement in fishing gear (Stanford et al., 2020; Gong et al., 2017). These issues have led to a notable decline in sea turtle populations (Mazaris et al., 2017; Stanford et al., 2020; Mazaris et al., 2023; Rivas et al., 2023). According to the IUCN Red List classification and criteria guidelines,^1^ six of the seven sea turtle species are currently listed as vulnerable, endangered, or critically endangered, and five of which could found in China, including the green turtles (*Chelonia mydas*), hawksbills (*Eretmochelys imbricata*), loggerheads (*Caretta caretta*), olive ridleys (*Lepidochelys olivacea*), and leatherbacks (*Dermochelys coriacea*) (Chan et al., 2009). According to the research, approximately 90% of the sea turtles in China inhabit the South Sea, and the estimated proportion of each species is approximately as follows: 87% for green turtles, 10% for hawksbills, and the remaining 3% is the combined proportion of leatherback turtles, loggerhead turtles, and olive ridley turtles (Chan et al., 2009; Mou et al., 2013). To alleviate some of the current pressures on sea turtles, global conservation efforts for sea turtles have started to take shape (Mazaris et al., 2017). The China Sea Turtle Conservation Action Plan (2019–2033) outlines a strategy to boost wild populations through captive breeding, to speed up the recovery of sea turtle numbers. However, releasing captive-bred turtles requires careful planning, particularly should avoid the release of turtles carrying pathogens, which could potentially pose a threat to the health, behavior and genetic diversity of the wild population (Kock et al., 2010). Thus, analyzing the microbial composition of captive sea turtles and assessing the presence of pathogens is crucial for effective turtle conservation and management strategies.

Advanced genomic technologies and bioinformatics approaches have exponentially increased the diversity of known microbes in recent years (Wensel et al., 2022; Forde and O’Toole, 2013; Ursell et al., 2012). The microbial community associated with the animal host plays a critical role in host development, physiology, immune response, metabolism, and reproduction, and may have an impact on the evolutionary success of the host (McFall-Ngai et al., 2013; Henry et al., 2021). Previous sea turtle microbiota studies have focused on the gastrointestinal tract (Abdelrhman et al., 2016; Chen et al., 2022; Kittle et al., 2018; Campos et al., 2018; Bloodgood et al., 2020; Arizza et al., 2019), with a small portion of the microbiota research found in the cloacal, oral, and nasal (Santoro et al., 2006; Zavala-Norzagaray et al., 2015; McNally et al., 2021; Price et al., 2017; Ahasan et al., 2017) regions. Additionally, most current research on the sea turtle microbiome relies on culture methods and 16S rRNA high-throughput sequencing, which limits our understanding of the functional aspects of the turtle microbiome. However, sea turtles have been proposed as health indicators of marine habitats and carriers of antibiotic resistant, for their longevity and migratory lifestyle (Blasi et al., 2020; Alduina et al., 2020). The spread of antibiotic resistance genes (ARGs) not only poses a threat to the original microbial communities in the marine environment, but also directly poses a threat to marine organisms, as there are multiple antibiotic-resistant bacteria (MDR bacteria) in the marine environment, which can cause harm to marine organisms. Moreover, these multiple antibiotic-resistant bacteria may serve as a platform for transmitting antibiotic resistance genes from the environment to humans (Drane et al., 2021). Furthermore, microbial virulence factors encompass a wide range of molecules produced by pathogenic microorganisms, enhancing their ability to evade their host defenses and cause disease. A deep understanding of the biological characteristics of microbial pathogens and their pathogenic factors is crucial for developing new therapeutic molecules and strategies to combat microbial infections (Leitão, 2020).

Therefore, we performed a detailed analysis of the oral, nasal, and cloacal microbiota composition and functional characteristics in three species of sea turtles, including green turtles (*Chelonia mydas*), loggerheads (*Caretta caretta*), and hawksbills (*Eretmochelys imbricata*), using shotgun metagenomic sequencing. Our objectives are (1) to investigate the microbial composition and identify the potential pathogens; (2) to discover the functional genes including ARGs and virulence factors, aiming to provide basic knowledge for the work related to assessing the potential pathogenic risks of sea turtles, minimize interference with wild sea turtle populations, and protect the marine ecosystem, thereby further strengthening the efforts in sea turtle conservation.

## Materials and methods

### Sample source

All samples in our study were collected from the Hainan Province Sea Turtle Rescue and Conservation Center (18.5°N, 108.5°E), which is a provincial-level aquatic wildlife rescue institution established by the Hainan Provincial Department of Agriculture and Rural Affairs with the support of Hainan Tropical Marine University. It mainly receives live sea turtles confiscated by government-designated law enforcement departments. Since its establishment, it has received nearly 600 live sea turtles involved in cases from over a dozen provinces and cities across the country. At present, there are still 61 sea turtles remaining, which are mainly used for artificial breeding and scientific research. These turtles have been kept in captivity for 4 to 5 years. Most of them are in health status (i.e., without any disease symptoms or external injuries), and have not been treated with antibiotics. These turtles are separately placed in different ponds, with approximately two to three turtles in each pond. All the turtles share the circulating seawater. The main food of the turtles is fish, including squid and various other types of fish.

### Sample collection and DNA extraction

The samples were collected from August 20th to 24th, 2024. A total of 61 sea turtles were collected, including 48 green turtles, ten hawksbills, and three loggerheads. We documented essential information, including biomarkers, sex, and injury status, straight carapace length/width, weight, and the time of arrival at the rescue center of the sea turtles (Supplementary Table S1). For each turtle, oral, cloacal, and nasal swabs were obtained, with three swabs collected from each region. Before sample collection, sterile water was used to continuously flush around the sampling site until no obvious contaminants were present. Oral swab samples were collected by gently rotating a sterile dry cotton or synthetic swab over the mucous membranes of the tongue and palate. Cloacal and nasal samples were collected by inserting a swab approximately 10 cm into the respective cavity, applying gentle pressure in a circular motion, and rubbing the inner circumference two to three times (Ahasan et al., 2018; McNally et al., 2021; Trotta et al., 2021). The swab head was then broken off and placed in a sterile 5 mL. After collection, samples were immediately frozen using liquid nitrogen, transported to the laboratory, and stored at − 80 °C until DNA extraction.

Genomic DNA was extracted using the Qiagen AllPrep DNA/RNA Mini Kit (Qiagen, Germany) following the manufacturer’s guidelines. The concentration and quality of all DNA samples were evaluated using the NanoDrop-1000 spectrophotometer and agarose gel electrophoresis. To improve both the concentration and quality of the DNA, samples extracted from the same anatomical regions of two to four turtles were pooled, resulting in a total of 63 DNA samples prepared for subsequent sequencing. This mixing strategy was based on the premise that DNA samples are pooled from the same species of sea turtles, from the same anatomical region, of the same gender or with the same injury condition.

### Shotgun metagenomic sequencing and assembly

Genomic DNA was subjected to random fragmentation into segments of approximately 350 base pairs using a Covaris ultrasonic disruptor for library construction. Subsequently, DNA libraries were constructed, purified, analyzed, and quantified, followed by sequencing on an Illumina PE150 platform at Novogene in Beijing, China. The raw data generated from the Illumina sequencing platform were processed using Fastp^2^ to eliminate paired reads that exhibited adapter contamination, contained more than 10% ambiguous nucleotides, or had over a specified percentage of low-quality nucleotides (with base quality scores below 5). Bowtie2 software^3^ was employed with default parameters set to --end-to-end, --sensitive, -I 200, and -X 400 to filter out reads potentially originating from the host. For assembly analysis of the cleaned data, MEGAHIT software was utilized with assembly parameters set to --presets meta-large (--end-to-end, --sensitive, -I 200, -X 400) (Li et al., 2015). Scaffolds devoid of N were generated by segmenting the resulting scaffolds at the N junction.

### Gene prediction and taxonomy annotation

MetaGeneMark^4^ was employed to predict open reading frames (ORFs) for shafts of at least 500 base pairs from each sample, followed by filtering to exclude any predicted sequences shorter than 100 nucleotides. The CD-HIT software (Li and Godzik, 2006; Fu et al., 2012) was utilized to remove redundancy and generate a non-redundant initial gene catalog, defined as the nucleic acid sequences encoded by successive non-redundant genes. The following parameter settings were applied: -c 0.95, -G 0, -a 0.9, -g 1, -d 0. Clean data from each sample was then aligned to the initial gene catalog using Bowtie2 to quantify the number of reads corresponding to each gene in the sample alignment, with parameter settings of --end-to-end, --sensitive, -I 200, -x 400. Genes with read counts of two or fewer in each sample were excluded, resulting in a refined gene catalog (Unigenes) for further analysis.

DIAMOND software^5^ (Buchfink et al., 2015) was utilized to align unigene sequences with the Micro_NR database, which comprises sequences from bacteria, fungi, archaea, and viruses extracted from NCBI NR database.^6^ The alignment was conducted using the Blastp algorithm with a parameter setting of 1e-5. From the alignment results for each sequence, the one with a value <= min value *10 was selected. Since each sequence may yield multiple alignment results, the Lowest Common Ancestor (LCA) algorithm, implemented in the MEGAN software (Huson et al., 2007), was employed to determine the species annotation information for each sequence. Based on the results of the LCA annotation and the gene abundance table, the abundance of each sample at each taxonomic level, along with the corresponding gene abundance tables, was obtained.

In addition, it should be noted in advance that in 2021, the International Committee on Prokaryotic Taxonomy changed the classification names of 42 Prokaryotic phyla to be consistent with the International Nomenclature for Prokaryotes (Oren and Garrity, 2021). The new names are used for the phyla within this paper, such as *Pseudomonadota* (formerly *Proteobacteria*), *Bacteroidota* (formerly *Bacteroidetes*), *Bacillota* (formerly *Firmicutes*), *Actinomycetota* (formerly *Actinobacteria*), *Fusobacteriota* (formerly *Fusobacteria*), and *Spirochaetota* (formerly *Spirochaetes*).

### Functional annotation

DIAMOND software was utilized to align unigenes with entries in the functional database, using the following parameter settings: blast, -e 1e-5. The functional databases included the Kyoto Encyclopedia of Genes and Genomes (KEGG^7^) (Kanehisa et al., 2017), Nonsupervised Orthologous Groups (eggNOG^8^) (Huerta-Cepas et al., 2016), Carbohydrate-Active enZYmes (CAZy^9^) (Cantarel et al., 2009), and the Virulence Factor Databases (VFDB^10^). Based on the alignment results, the relative abundance at various functional levels was calculated (the relative abundance at each functional level is defined as the sum of the relative abundance of genes annotated at that functional level). Furthermore, unigenes were aligned with the Comprehensive Antibiotic Resistance Database (CARD^11^) (Jia et al., 2017) using the Resistance Gene Identifier (RGI) software (RGI built-in blast, default value < 1e-30) (McArthur et al., 2013). Utilizing the RGI alignment result and unigenes abundance information, the relative abundance of each Antimicrobial Resistance Ontology (ARO) was determined.

### Statistical analysis

Based on the computations outlined above, we obtained several datasets, including an overview gene catalog and a table detailing microbiome composition and functional abundance at each taxonomic level. Utilizing these data, we analyzed the diversity and differences in microbiome composition and function across various samples, turtle species (green turtles, hawksbills, and loggerheads), and tissue region (oral, nasal, and cloacal). We used two alpha diversity indexes (Chao 1 and Shannon diversity index) to assess the community richness and diversity. The Wilcoxon rank sum test was used to examine the pairwise differences among three turtle species and tissue regions, respectively. We used principal coordinate analysis (PCoA) based on Bray–Curtis distance to visualize sample clustering patterns. Furthermore, we used the adonis function in vegan package to perform the permutational multivariate analysis of variance (PERMANOVA, permutation = 9,999) to examine the effects of host species and tissue regions on microbiota. Additionally, we performed the ANOSIM analysis to examine the differences between and within groups. The Kruskal-Wallis test was used to detect taxa and function with differences in abundance among different groups. The linear discriminant analysis (*LDA*) effect size (LEfSe) (Segata et al., 2011) was performed to obtain the biomarker bacterial with *LDA* > 3, *p* < 0.05 in each host species and tissue regions. And we used statistical analysis of metagenomic profiles (STAMP) (Parks et al., 2014) to visualize the top 20 differential functions among different groups. Furthermore, we analyzed the pathogens associated with these marine reptiles to evaluate the potential pathogenic risks and enhance the survival rates of sea turtles. We utilized the human pathogen lists (Bartlett et al., 2022), which enumerates 1,513 bacterial pathogens known to affect humans before 2021, comprising 1,100 established and 403 putative pathogens. These pathogens were classified into 327 genera. Additionally, we incorporated data from the BacDive database,^12^ encompassing 2,292 bacterial strain pathogens affecting animals, 1,611 affecting humans, and 384 affecting plants, linked to 809 species and 210 genera. By integrating these two data sources, we identified a total of 412 pathogen genera and 2,134 pathogen species.

## Results

### Sea turtle microbiome gene catalog constructed using metagenomic analysis

The shotgun metagenomic sequencing produced a total of 705.81 gigabytes (GB) of raw Illumina data from 63 genomic DNA. After quality control, 697.98 Gb of clean, high-quality data remained, achieving an effective quality control rate of about 98.89% (Supplementary Table S2). The metagenomic assembly and open reading frame (ORF) prediction yielded 24.50 million contigs and 43.37 million ORFs (Supplementary Table S2). Ultimately, a nonredundant sea turtle microbial gene catalog (STMGC) was created, containing 10,733,232 unique genes after de-redundancy of the ORFs. To assess the validity and completeness of the identified genes, a rarefaction analysis was conducted with 50 random samples, and the core-pan gene rarefaction curve approached a plateau (Supplementary Figures S1A,B). A heatmap was generated to analyze the correlations between samples based on the gene table (Supplementary Figure S1C). A box plot illustrating the differences in gene numbers among groups revealed that the oral region had the highest gene counts, followed by the nasal and the cloacal across three turtle species (Supplementary Figure S1D).

### Overview of the sea turtle microbiome taxonomic composition

**Figure 1:**
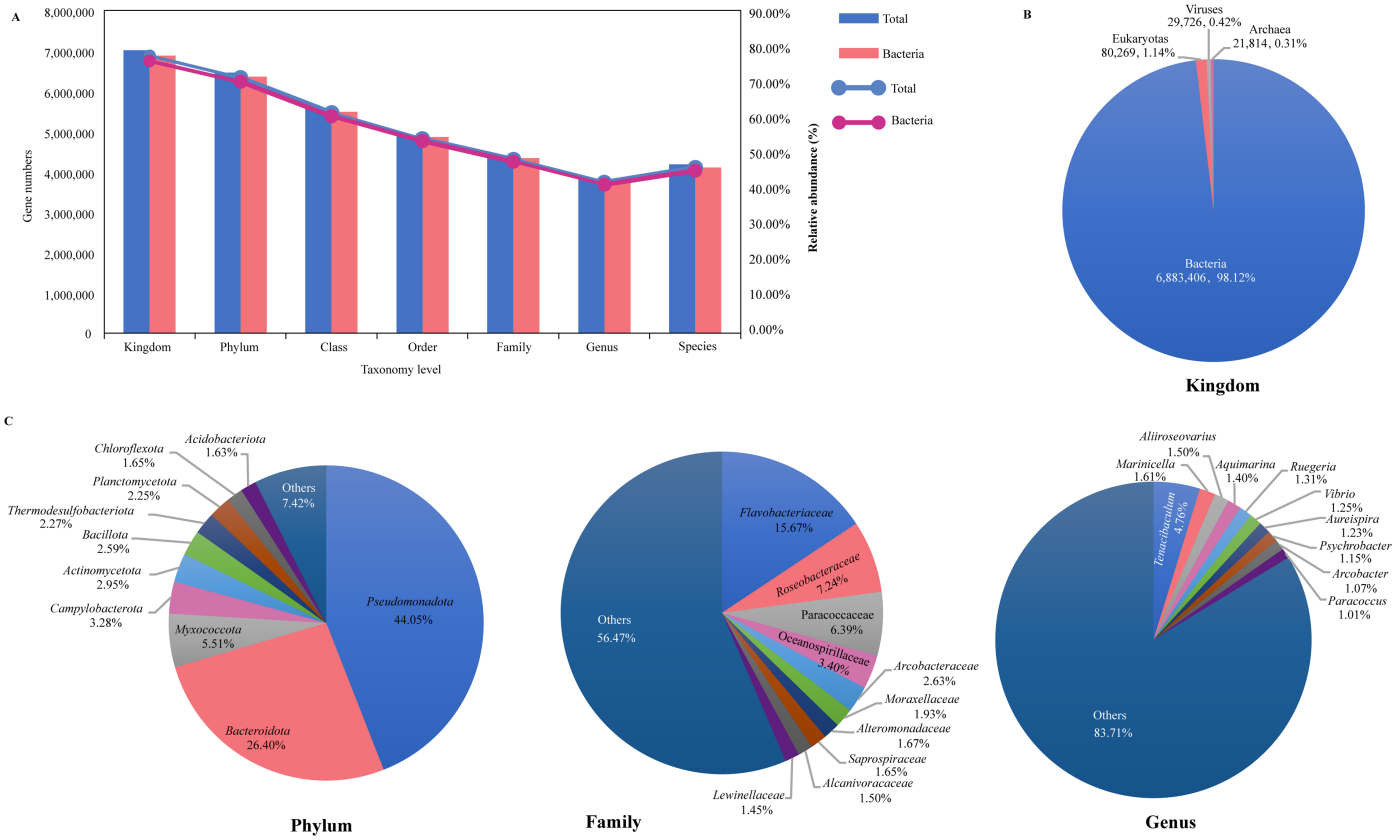
Overview of the sea turtle microbiome taxonomic composition. **(A)** The gene counts and relative abundance of different taxonomic levels (from kingdom to species level). Blue represents total taxonomic taxa, and pink represents bacterial taxa. **(B)** The relative abundance of bacteria, eukaryotes, viruses, and archaea. **(C)** The relative abundance of the top 10 bacteria at phylum, family, and genus level.

A total of 9,067,768 (84.48%) nonredundant genes were annotated in the NCBI NR database (Supplementary Table S3). As shown in Figure 1A, from kingdom to species level, there are, respectively, annotated in these NR genes at 77.36% (7,015,215), 71.14% (6,450,983), 61.41% (5,568,232), 54.16% (4,910,861), 48.40% (4,388,677), 42.03% (3,811,134), and 46.11% (4,181,434). At the kingdom level, 6,883,406 (98.12%) genes were identified as bacteria, while eukaryotes accounted for only 80,269 genes (1.14%), viruses for 29,726 genes (0.42%), and archaea for 21,814 genes (0.31%) (Figure 1B). Our research primarily concentrated on the bacterial component, which constituted 98.12% of the microbial community. For the bacterial, we identified 165 phyla (including 45 non-Candidatus and 120 Candidatus phyla, total 6,352,686 genes), 145 classes (5,486,773 genes), 315 orders (4,858,058 genes), 782 families (4,337,212 genes), 3,922 genera (3,758,642 genes), and 36,709 species (4,107,141 genes) of microorganisms (Figure 1A). At the phylum level, the microbiome of sea turtles was predominantly composed of *Pseudomonadota* (formerly *Proteobacteria*, 44.05%) and *Bacteroidota* (formerly *Bacteroidetes*, 26.40%), which together made up 70.45% of the total. Other prevalent phyla included *Myxococcota* (5.51%), *Campylobacterota* (3.28%), *Actinomycetota* (formerly *Actinobacteria*, 2.95%), *Bacillota* (formerly *Firmicutes*, 2.59%), *Thermodesulfobacteriota* (2.27%), *Planctomycetota* (2.25%), *Chloroflexota* (1.65%), and *Acidobacteriota* (1.63%) (Figure 1C, phylum). At the genus level, the most ten abundant bacteria were *Tenacibaculum* (*Flavobacteriaceae* of *Bacteroidota*), *Marinicella* (*Alcanivoracaceae* of *Campylobacterota*), *Aliiroseo*var*ius* (*Paracoccaceae* of *Pseudomonadota*), *Aquimarina* (*Flavobacteriaceae* of *Bacteroidota*), *Ruegeria* (*Roseobacteraceae* of *Pseudomonadota*), *Vibrio* (*Vibrionaceae* of *Pseudomonadota*), *Aureispira* (*Saprospiraceae* of *Bacteroidota*), *Psychrobacter* (*Moraxellaceae* of *Pseudomonadota*), *Arcobacter* (*Arcobacteraceae* of *Campylobacterota*), and *Paracoccus* (*Paracoccaceae* of *Pseudomonadota*), collectively representing 16.29% of the total counts (Figure 1C, genus). Additionally, we examined the microbial composition at various taxonomic levels across all samples, finding that *Pseudomonadota* and *Bacteroidota* were the most prevalent phyla, while the distribution of other phyla varied in different samples (Supplementary Figure S2A). These differences were also apparent at other taxonomic levels, suggesting that microbiomes may vary among different turtle species and tissue regions (Supplementary Figure S2).

### Diversity analysis of the sea turtle microbiome

**Figure 2:**
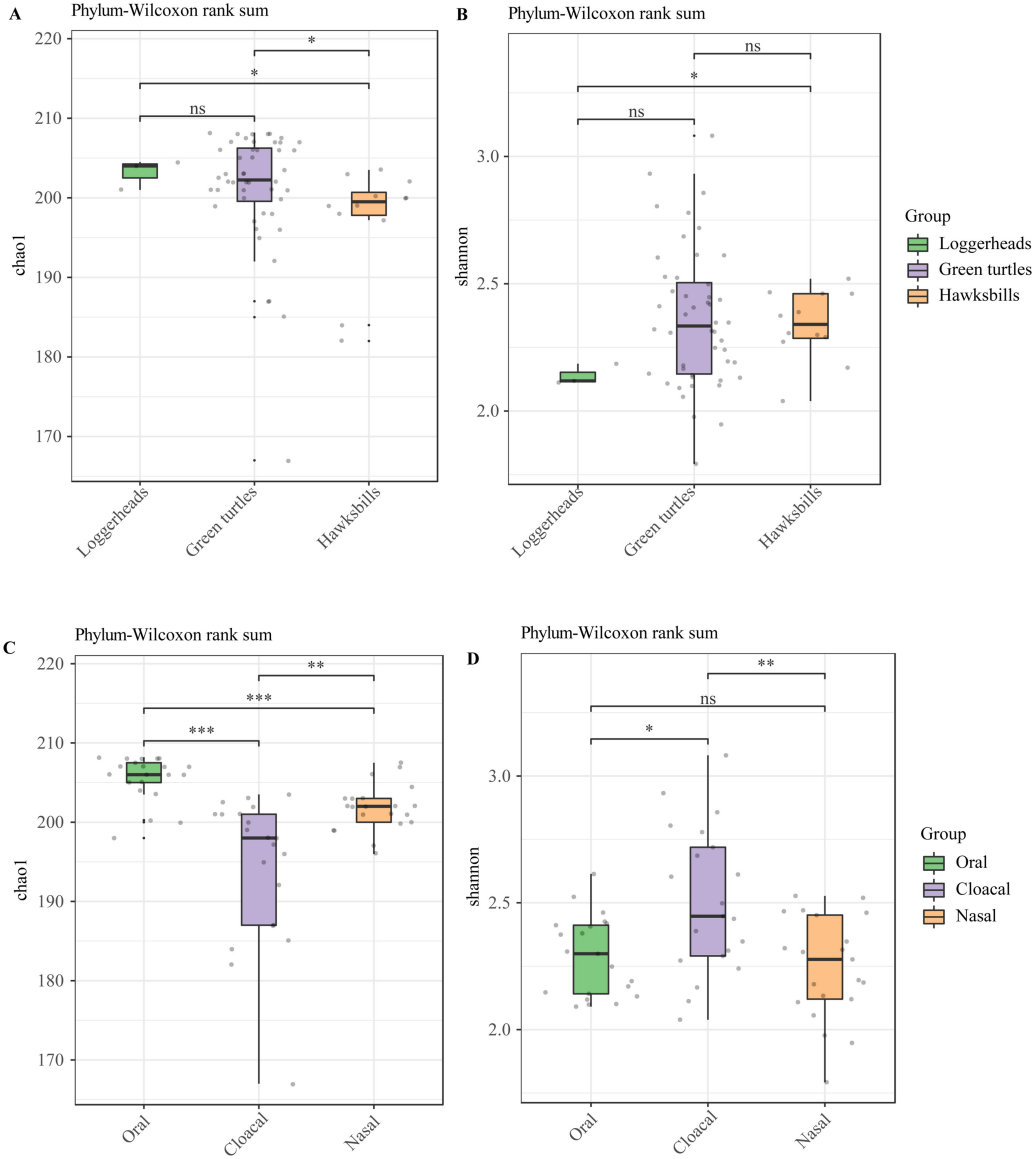
The alpha diversity analysis of the sea turtle bacterial community at the phylum level based on host species and tissue region. **(A,B)** The Chao 1 index and Shannon diversity index across three host species. **(C,D)** The Chao 1 index and Shannon diversity index across three tissue regions. *p*-values indicate the confidence level of statistical analyses, with *p* < 0.05 indicating statistically significant differences. ns represents no significant differences, * represents *p* < 0.05, **represents *p* < 0.01, and ***represents *p* < 0.001.

The alpha diversity analysis showed that there were no significant differences in loggerheads and green turtles using Chao 1 and Shannon index, but a significant difference was noted between loggerheads and hawksbills at the phylum and order levels when using the Shannon index (Figures 2A,B; Supplementary Figures S3A,B). Furthermore, significant differences were observed between green turtles and hawksbills at the phylum level when using the Chao 1 index (Figure 2A). In terms of tissue region, the Wilcoxon test applied to the Chao 1 index showed significant differences across all taxonomic levels at the three tissue regions (Figure 2C; Supplementary Figure S3C). Additionally, significant differences in the Shannon index between oral and cloacal samples were found at all taxonomic levels (Figure 2D; Supplementary Figure S3D).

**Figure 3:**
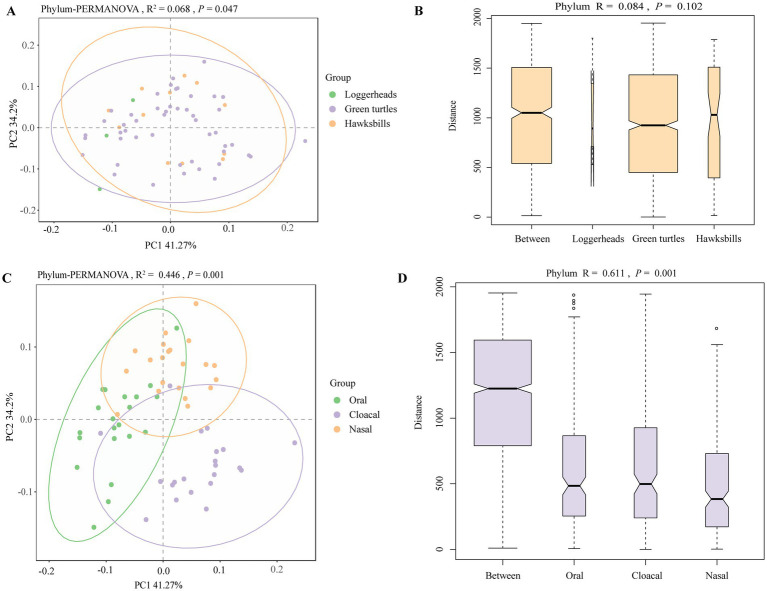
The beta diversity analysis of the sea turtle bacterial community at the phylum level based on host species and tissue region. **(A,C)** The PCoA results of bacteria at the phylum level based on three turtle species and tissue regions. **(B,D)** ANOSIM results of bacteria at the phylum level based on three turtle species and tissue regions.

Principal coordinates analysis (PCoA) clearly distinguished tissue regions, while the differences among host species were less marked (Figures 3A,C; Supplementary Figures S4A,B). PERMANOVA analysis revealed significant statistical differences in microbial profiles across the three species and tissue regions at the phylum level, accounting for 6.8% (*R*^2^ = 0.068, *p* = 0.047) and 44.6% (*R*^2^ = 0.446, *p* = 0.001) of the variation, respectively (Figures 3A,C). This pattern was also evident at other taxonomic levels (Supplementary Figures S4A,B). Pairwise comparisons of the three sea turtles indicated that the differences between loggerheads and hawksbills were not statistically significant at any taxonomic level, while significant differences were found between loggerheads and green turtles at the order and species levels (Supplementary Table S4). Additionally, significant differences were noted between green turtles and hawksbills from the order to species levels. In the analysis of tissue region, all comparisons from phylum to species levels showed significant differences (Supplementary Table S4). ANOSIM analysis further confirmed significant differences among the three tissue regions at all taxonomic levels (Figures 3B,D; Supplementary Figures S4C,D). When comparing same tissue region from different species, PCoA results revealed that the same tissue regions of different turtle species always clustered together, and the cloacal was clearly separated from the oral and nasal regions (Supplementary Figure S5). Pairwise comparisons showed that there were significant differences in the same tissue regions between green turtles and hawksbill from class to species levels (Supplementary Table S4).

### Major differences in the sea turtle microbiome based on host species and tissue region

To investigate the major differences in the sea turtle microbiome, we conducted correlation analyses referring to the host species and tissue region, respectively. The comparative analysis of bacterial composition across various taxonomic levels highlighted both similarities and differences among host species and tissue regions, respectively (Supplementary Figures S6A,B). Within the three host species, green turtles had more unique bacterial species. In three tissue regions, oral samples contained a greater number of distinct species (Supplementary Figures S6C,D).

**Figure 4:**
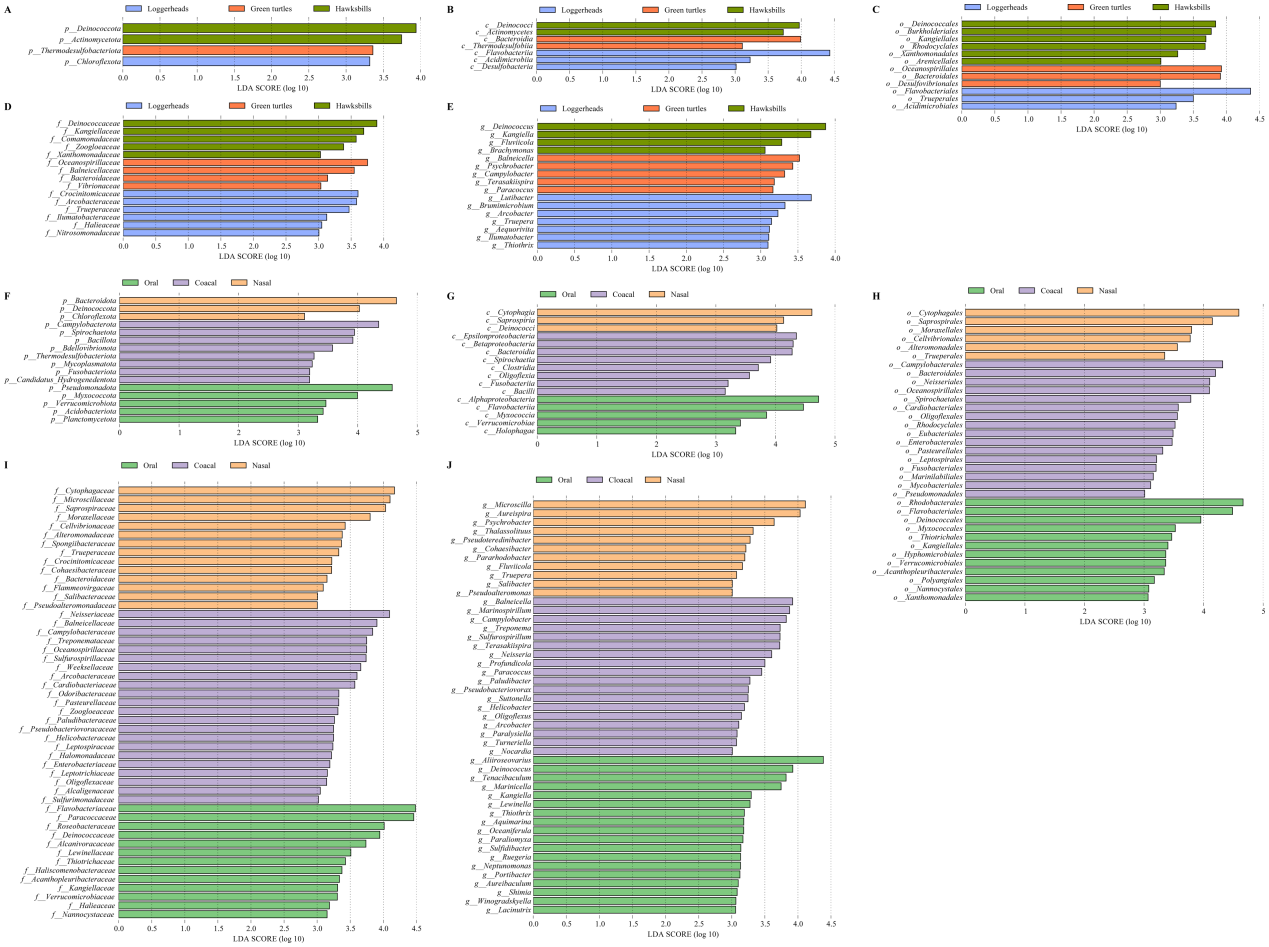
The LEfSe analysis of the sea turtle bacterial community based on turtle species and tissue region. **(A–E)** The differential bacteria from phylum to genus level among the three turtle species. **(F–J)** The differential bacteria from phylum to genus level among the three tissue regions.

We identified a total of 54 bacterial taxa that were differentially abundant across the three host species by linear discriminant analysis (Figures 4A–E). Notably, *Deinococcota* and *Actinomycetota* were more prevalent in hawksbills, while *Thermodesulfobacteriota* and *Chloroflexota* were more abundant in green turtles and loggerheads, respectively, at the phylum level (*LDA* > 3, *p* < 0.05; Figure 4A). At the class level, the most differentially abundant taxa in hawksbills were identified as *Deinococci* and *Actinomycetes*, whereas *Bacteroidia* and *Thermodesulfobiia* were predominant in green turtles, and *Flavobacteriia*, *Acidimicrobiia*, and *Desulfobacteria* were more abundant in loggerheads (Figure 4B). At the order level, hawksbills exhibited six significantly abundant bacterial orders, whereas green turtles and loggerheads displayed three each (Figure 4C). The number of significantly abundant families varied, with five in hawksbills, four in green turtles, and six in loggerheads (Figure 4D). The significantly abundant genera in hawksbills included *Deinococcus* within the *Deinococcota* and *Kangiella* within the *Pseudomonadota*. The significantly abundant genera comprised *Balneicella*, *Psychrobacter*, *Campylobacter*, *Terasakiispira*, and *Paracoccus* in green turtles, while loggerhead exhibited *Lutibacter*, *Arcobacter*, and five more (Figure 4E).

In the analysis of tissue region, we identified 16, 16, 34, 49, and 47 differentially abundant bacterial taxa from phylum to genus level (*LDA* > 3). Cloacal samples had the highest number of differentially abundant bacteria (72), followed by oral samples (53) and nasal samples (37) (Figures 4F–J). *Pseudomonadota* was significantly more abundant in oral samples (*LDA* = 4.58), and *Bacteroidota* were present a greater abundance in nasal samples (*LDA* = 4.65) (Figure 4F). Additionally, *Myxococcota*, *Verrucomicrobiota*, *Acidobacteriota*, and *Planctomycetota* were found to be more abundant in oral samples, while *Deinococcota* and *Chloroflexota* were significantly abundant in nasal samples. In cloacal samples, *Campylobacterota* was identified as the most significantly abundant phylum (*LDA* = 4.35), followed by *Spirochaetota*, *Bacillota*, *Bdellovibrionota, Thermodesulfobacteriota*, *Mycoplasmatota*, *Fusobacteriota*, and *Candidatus Hydrogenedentota* (Figure 4F). The differential patterns observed at the phylum level were consistent across other taxonomic levels. For instance, differentially abundant bacteria in oral samples included *Alphaproteobacteria*, five orders (*Rhodobacterales*, *LDA* > 4), six families (*Paracoccaceae, Roseobacteraceae*, *LDA* > 4), and seven genera (*Aliiroseovarius*, *LDA* > 4) within *Pseudomonadota*, as well as *Myxococcia*, three orders, *Nannocystaceae*, and *Paraliomyxa* in *Myxococcota*, along with various taxa in *Verrucomicrobiota* and *Acidobacteriota*. The nasal samples contained two classes, two orders, seven families, and four genera within *Bacteroidota*. The cloacal samples included *Epsilonproteobacteria*, *Campylobacterales*, five families, and four genera (Campylobacter, Arcobacter) within *Campylobacterota*, along with seven bacteria in *Spirochaetota*, 13 bacteria in *Bacillota*, and six bacteria in *Bdellovibrionota* (Figures 4G–J).

### Potential pathogenic bacteria detected in the sea turtle microbiome

**Figure 5:**
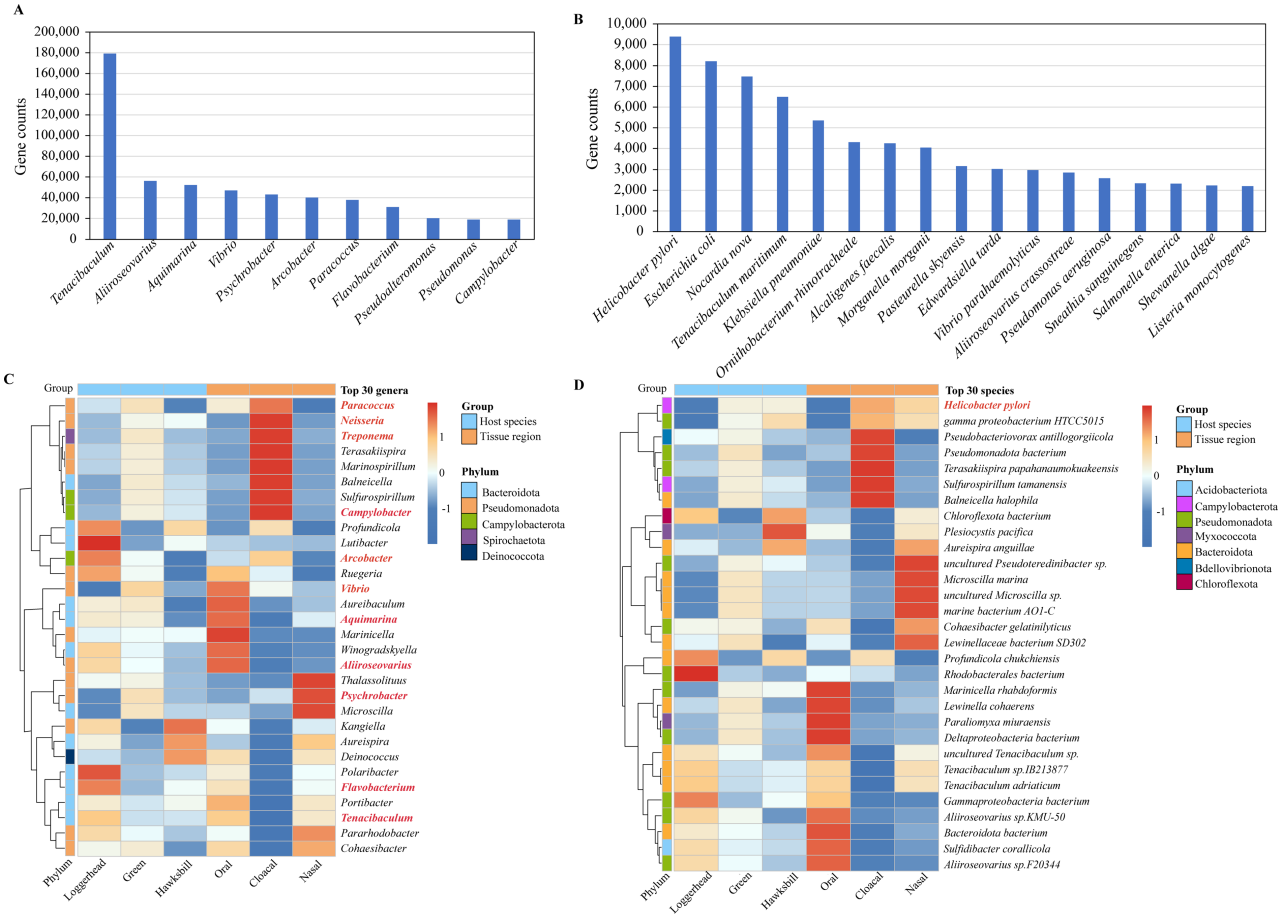
The potential pathogens detected in sea turtle microbiomes. **(A)** The potential pathogenic genera with gene counts exceeding 0.5%. **(B)** The potential pathogenic species with gene counts greater than 0.05%. **(C)** The pathogenic genera detected in the top 30 genera. The red mark is a pathogenic genus. **(D)** The pathogenic species detected in the top 30 species. The red mark is a pathogenic species.

Our investigation ultimately identified 389 pathogen genera and 1,445 pathogen species, with total gene counts of 25.24% and 3.85%, respectively (Supplementary Table S5). Notably, 11 genera exhibited gene counts exceeding 0.5%, including *Tenacibaculum*, *Aliiroseovarius*, *Aquimarina*, *Vibrio*, *Psychrobacter*, *Arcobacter*, *Paracoccus*, *Flavobacterium*, *Pseudoalteromonas*, *Pseudomonas*, and *Campylobacter* (Figure 5A). Additionally, 17 species demonstrated gene counts greater than 0.05%, such as *Helicobacter pylori*, *Escherichia coli*, *Nocardia nova*, *Tenacibaculum maritimum*, *Klebsiella pneumoniae*, *Ornithobacterium rhinotracheale*, *Alcaligenes faecalis*, *Morganella morganii*, *Pasteurella skyensis*, *Edwardsiella tarda*, *Vibrio parahaemolyticus*, *Aliiroseovarius crassostreae*, *Pseudomonas aeruginosa*, *Sneathia sanguinegens*, *Salmonella enterica*, *Shewanella algae*, and *Listeria monocytogenes* (Figure 5B).

Among the top 30 genera identified across all samples, 11 were classified as pathogenic bacterial genera, as indicated in red in Figure 5C. Of these 11 pathogen genera, with three turtles, *Paracoccus*, *Treponema*, *Campylobacter*, *Vibrio*, and *Psychrobacter* were found to be more abundant in green turtles, while *Arcobacter*, *Aliiroseovarius*, *Flavobacterium*, and *Tenacibaculum* were more prevalent in loggerheads. With three tissue regions, *Paracoccus*, *Neisseria*, *Treponema*, *Campylobacter*, and *Arcobacter* were predominantly detected in cloacal samples, while *Vibrio*, *Aquimarina*, and *Aliiroseovarius* were more abundant in oral samples, and *Psychrobacter* was more abundant in nasal samples (Figure 5C). Among the top 30 species identified in all samples, only *H. pylori* was identified as a pathogenic bacterium. Among the three turtles, it is mainly found in green turtles and hawksbills, while in the three tissue regions, it is mainly present in the cloacal samples, followed by nasal samples (Figure 5D).

### Functional characteristics of the sea turtle microbiome

The functional gene annotation results showed that the KEGG, eggNOG and CAZY databases, respectively, annotated 29.35% (3,150,254), 76.11% (8,169,033) and 3.73% (400,661) of the non-redundant genes. Through the CARD and VFDB databases, 1,904 and 344,839 genes were annotated, and 157 antibiotic resistance genes (ARGs) and 697 virulence genes were discovered (Supplementary Table S3; Supplementary Figure S7A).

An analysis of KEGG metabolic pathways revealed that microbial functions were predominantly linked to metabolic processes, with a particular emphasis on “Amino acid metabolism” and “Carbohydrate metabolism” (Supplementary Figure S7B). In the Clusters of Orthologous Groups (COG) database, “Amino acid transport and metabolism” emerged as the most enriched functional category, aside from the “Function unknown” category (Supplementary Figure S7C). In the CAZY database, the “Glycosyl Transferases” category was found to be significantly enriched (Supplementary Figure S7D). Furthermore, the CARD and VFDB databases identified “Antibiotic Efflux” and “Immune Modulation” as enriched functions, respectively (Supplementary Figures S7E,F). To illustrate the overall distribution of functions across different host species and tissue region, the top 20 most abundant results were selected and represented in bar plots (Supplementary Figures S8A–H).

**Figure 6:**
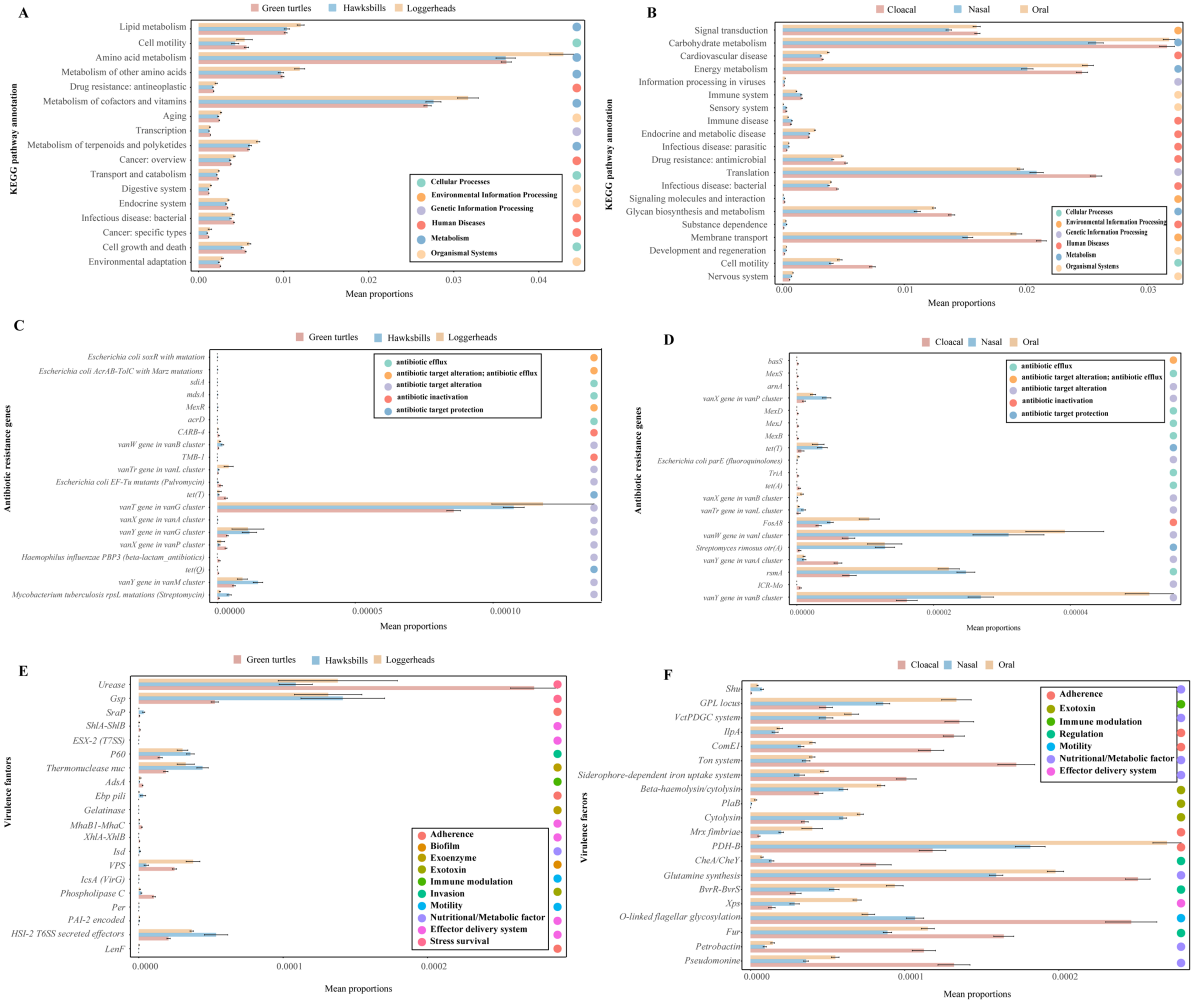
The differential functions of the microbiome across three turtle species and tissue regions by STAMP analysis. **(A,B)** The top 20 differential functions of KO pathways across three turtle species and tissue regions, respectively. **(C,D)** The top 20 differential ARGs across turtle species and tissue regions. **(E,F)** The top 20 differential virulence factors across turtle species and tissue regions.

Additionally, we employed STAMP analysis to visualize the top 20 differential functions of KO pathways, antibiotic resistance genes (ARGs), and virulence factors across three host species and tissue regions (Figure 6). Across three host species, a total of 17 KO pathways exhibited significantly differential abundance, with seven of these pathways ranking among the top 20 most abundant. Notably, functions related to metabolism, such as “metabolism of terpenoids and polyketides,” “Metabolism of cofactors and vitamins,” “Metabolism of other amino acids,” “Amino acid metabolism,” and “Lipid metabolism” were found to be more abundant in loggerheads (Figure 6A). In comparison to tissue region, eight of the top 20 abundant KOs functions demonstrated significantly differential abundance, with “Cell motility,” “Membrane transport,” “Glycan biosynthesis and metabolism,” “Infection disease: bacterial,” “Translation,” and “Drug resistance: antimicrobial” being significantly more prevalent in cloacal samples (Figure 6B).

Furthermore, we analyzed the differences in ARGs and virulence factors across three host species and tissue regions. Notably, 55 ARGs and 535 virulence factors were identified as common among the three host species, while 81 ARGs and 597 virulence factors were shared across the three tissue regions (Supplementary Figures S8I–L). The green turtles and cloacal samples contained more unique ARGs and virulence genes. The top 20 abundant ARGs and virulence factors were consistently found across all samples, and the top 20 ARGs exhibited higher abundance in hawksbills and oral samples (Supplementary Figures S8E,F), whereas the top 20 virulence factors were more abundant in loggerheads and cloacal samples (Supplementary Figures S8G,H). Of the top 20 differential abundance ARGs identified in three host species, 12 ARGs were common across all host species but most of them had a lower abundance in green turtles, while eight ARGs were not detected in loggerheads, including the *vanX* gene in the *vanA* cluster, *TMB-1*, *acrD*, *MexR*, *sdiA*, *mdsA*, *E. coli AcrAB-TolC* with *MarR* mutations conferring resistance to ciprofloxacin and tetracycline, and *E. coli soxR* with mutation conferring antibiotic resistance (Figure 6C). When comparing the three tissue regions, 14 ARGs were common in three tissue regions and most of them had lower abundance in cloacal samples, while six ARGs were not detected in nasal samples, including *TriA*, *MexB*, *MexJ*, *MexD*, *MexD*, and *basS* (Figure 6D). In an analysis of virulence factors, 14 of the top 20 differential abundance virulence factors were shared among three host species, and six were not detected in green turtles, such as *LenF*, *Per*, *IcsA (VirG)*, *Gelatinase*, *Ebp pili*, and *ESX-2* (T7SS) (Figure 6E). In comparing across tissue region, 11 virulence factors were significantly more abundant in cloacal samples, such as *Pseudomonine*, *Petrobactin*, *Fur*, O-linked flagellar glycosylation, Glutamine synthesis, *CheA / CheY,* Siderophore-dependent iron uptake system, Ton system, *ComE1*, *IlpA*, and *VctPDGC* system. Conversely, eight virulence factors were found to be more abundant in oral samples, including *Xps*, *BvrR-BvrS*, *PDH-B*, *Mrx* fimbriae, cytolysin, *PlaB*, Beta-haemolysin/cytolysin, and *GPL locus* (Figure 6F).

## Discussion

Sea turtles are regarded as “flagship” and “umbrella” species in marine ecosystems, appreciated for their ecological importance and vital role in preserving biodiversity and assessing ecosystem health (Senko et al., 2022; Lovich et al., 2018; Bouchard and Bjorndal, 2000), but they are seriously endangered due to factors mainly related to human activities and climate change such as pollution, temperature increase, and predation (Gong et al., 2017; Stanford et al., 2020). Infectious and parasitic diseases may contribute to reducing the number of sea turtles. Bacteria are widespread in marine environments, and depending on the species, may act as primary or opportunistic pathogens. Most of them can infect other animal species, including humans, and may cause mild or severe diseases. Therefore, any direct or indirect contact between humans and sea turtles, their products, and environment where they live represent a One Health threat (Mashkour et al., 2020). Our study is the first to combine the oral, nasal, and cloacal of green turtles, loggerheads, and hawksbills for comparative analysis. The objective is to investigate the oral, nasal, and cloacal microbiota composition and functional characteristics of three threatened sea turtles, intending to gain knowledge and understanding to improve husbandry in rehabilitation settings, improve treatment and prevention of disease processes, and increase the survival rate of sea turtles. In addition, we hope to identify the microorganisms that could serve as potential human pathogens, such as multidrug-resistant bacteria, to provide theoretical knowledge, support for potential turtle and human health, disease prevention and treatment, and promote future conservation efforts.

Our results suggested that the *Pseudomonadota* was the most abundant phylum across all samples. There was no significant difference in its abundance among the three species, but in the three tissue regions, it was significantly higher in oral samples. In 2020, Scheelings et al. have reported microbiota data obtained from wild nesting females of all seven extant sea turtle species via distal colon swab, and their results showed that the *Proteobacteria* was the predominant bacterial phylum across all sea turtle species, which is consistent with our results (Scheelings et al., 2020). Some previous studies have also reported that *Proteobacteria* was the dominant phylum in stranded sea turtles, such as loggerheads of Tuscany and Liguria regions and green turtles of Great Barrier Reef regions (Abdelrhman et al., 2016; Ahasan et al., 2017; Ahasan et al., 2018). Additionally, Campos et al. and Samuelson et al. noted that a significant increase in the abundance of *Proteobacteria* was associated with prolonged captivity and treatment with antibiotics (Campos et al., 2018; Samuelson et al., 2020). Campos et al. and Bloodgood et al. also have found that sea turtles which consumed more animal protein had a higher relative abundance of the *Proteobacteria* (Bloodgood et al., 2020; Campos et al., 2018). These studies suggest that in our research, the *Proteobacteria* was dominant phylum among all sea turtles, possibly because sea turtles have been in captivity and consuming seafood for a long period. Furthermore, previous studies have shown that the high prevalence of *Proteobacteria* is one of the recognized signatures of dysbiosis as well as an indication of disease in animals, including humans (Shin et al., 2015). Our study demonstrated that among the 389 potential pathogenic genera detected, 167 belong to the *Proteobacteria*, particularly *Aliiroseo*var*ius*, *Vibrio*, *Psychrobacter*, *Paracoccus*, and *Neisseria*, which are affiliated with the top 30 abundant genera. *Aliiroseovarius crassostreae*, formerly known as *Roseovarius crassostreae*, is the causative agent of *Roseovarius* oyster disease (ROD), which results in high mortality rates in hatchery-raised juvenile eastern oysters in the northeast United States (Kessner et al., 2016). The abundance was significantly higher in oral samples, which may be because the diet origin of turtles was fish, and they might have been exposed to shellfish in the breeding facility and thus contracted the infection. Additionally, sea turtles have been frequently identified as carriers of *Vibrio* spp. (Zavala-Norzagaray et al., 2015; Fichi et al., 2016; Andrews et al., 2003), becoming potential sources of infections for humans. *Vibrio* spp. has been isolated from lesions of sea turtles of different species, including olive ridley sea turtles, green turtles, and loggerheads (Acuña et al., 1999; Fichi et al., 2016; Trotta et al., 2021). Although the *Vibrio* spp. naturally present in marine ecosystems and estuarine waters, indicating these bacteria may be some that coexist with the environment and sea turtles (Baker-Austin et al., 2018). However, among *Vibrio* spp., 12 species can cause infections in humans. These bacteria pose potential risks to the health of sea turtles and human.

In contrast, some studies have reported that *Firmicutes* was the dominant phylum, whether wild or stranded sea turtles, especially in green turtles (Abdelrhman et al., 2016; Price et al., 2017; Ahasan et al., 2017; Arizza et al., 2019; Chen et al., 2022). This may be due to the key role of *Firmicutes* in the digestion of complex polysaccharides (Hong et al., 2011). However, in our study, the abundance of the *Firmicutes* was relatively lower, which was similar to the research of Scheelings et al. (2020). They found that the abundance of the *Firmicutes* in green turtles and loggerheads was the lowest among all sea turtle species, suggesting that this phylum may not be as important for cellulose digestion in herbivorous reptiles as previously reported. However, considering that all the female sea turtles in their investigation were likely fasting for a long period, which led them to believe that this might have changed the bacterial phylum that is very important for digestive function, thereby affecting the turtle microbiota. Also, there are numerous examples in vertebrates, both terrestrial and aquatic, which indicate that the abundance of the *Firmicutes* is lower than that of other phyla in herbivorous species (Hong et al., 2011; Ishaq and Wright, 2012; Pope et al., 2012; Sullam et al., 2012). Therefore, considering that the microbiota of sea turtles may be affected by factors such as species, health conditions, captivity, and feeding situations, we should view these results with caution.

The *Bacteroidota* (formerly *Bacteroidetes*) was another dominant phylum across all samples. LEfSe analysis revealed no significant difference in its abundance among the three host species, but considering the three tissue regions, its abundance was significantly higher in nasal samples. Previous studies on the microbiota of sea turtles have shown that *Bacteroidota* is always a core bacterial phylum in sea turtles (Campos et al., 2018; Ahasan et al., 2018; Ahasan et al., 2017; Abdelrhman et al., 2016; Al-Bahry et al., 2009). Part of the reason might be that the members of the *Bacteroides* possess a variety of genes encoding carbohydrate activity, enabling them to easily switch between different energy sources through complex regulatory mechanisms based on the availability of different energy sources in the gastrointestinal tract (Xu et al., 2007; Thomas et al., 2011). Another reason of the decrease in abundance of *Firmicutes* and increase in *Bacteroidetes* is likely because of abundant seafood. Previous studies have shown that *Bacteroidetes* consists of many bile-tolerant organisms that aid in protein digestion, and, in humans that switched from a fiber-rich diet to an animal protein-based diet, there was a decrease in *Firmicutes* and an increase in *Bacteroidetes* in as little as 4 days (Wu et al., 2011; David et al., 2014). However, its specific functions in the nasal and oral of sea turtles remain to be further studied. Notably, the members of *Campylobacterota* were significantly higher in cloacal samples in our study, particularly *Arcobacteriaceae*, *Campylobacteraceae*, and *Helicobacteraceae*, which have recently been found to form unique, cold-adapted communities in ectothermic reptiles (Gilbert et al., 2019). Previous studies have also reported that loggerhead cloacal communities are a promising source of novel *Campylobacterota* (Filek et al., 2024). In our study, *Campylobacter* was significantly more abundant in green turtles. This might be because the number of green sea turtles in our sample was the highest, while the number of loggerheads was the lowest. *Campylobacterota* is also recognized as an important human pathogen, and half of the human population is colonized with the ulcer-causing stomach bacterium *H. pylori* (Eusebi et al., 2014). The *H. pylori* is the most abundant pathogenic species detected in our study. It reminds us to pay more attention to the intestinal problems of sea turtles.

In terms of the functions of the sea turtle microbiome, three turtle species and tissue regions displayed partly similar patterns and partly different features, which are consistent with the microbiome composition patterns. For example, the most abundant KEGG pathways and CAZymes across all samples were linked to metabolic processes and glycosyl transferases, which are similar with previous study on gut communities between hawksbills and green turtles, suggesting that sea turtles may hydrolyze and ferment complex carbohydrates or polysaccharides in the food by utilizing the enzymes expressed by gut microbes (Chen et al., 2022). Additionally, it is worth our attention that previous studies have discovered resistant bacteria in the gut microbes of wild loggerheads and green turtles (Alduina et al., 2020; Blasi et al., 2020; Conrad et al., 2023). And Yu et al. have discovered that the ARGs are significantly higher in artificially bred green turtles than in wild turtles (Niu et al., 2024). In our study, the glycopeptide and tetracycline resistance genes were dominant ARGs, and a higher abundance of ARGs was detected in hawksbills and oral samples, which is consistent with the study of Chen et al. (2022). Glycopeptide antibiotics are frequently used to treat life-threatening infections caused by multidrug-resistant Gram-positive pathogens, such as *Staphylococcus aureus*, *Enterococcus* spp., and *Clostridium difficile* (Marcone et al., 2018). Tetracycline antibiotics have been widely detected in terrestrial and aquatic environments (Chen et al., 2011; Li et al., 2011). Considering that the spread of drug-resistant microorganisms can occur through direct contact or the food chain, the large number of antibiotic resistance genes (ARGs) present in the sea turtles and their oral samples may mainly result from the fact that the sea turtles in this study mainly feed on farmed fish and have lived in recirculating seawater for a long time. This might introduce drug-resistant microorganisms into the microbial communities of the sea turtles. Furthermore, virulence genes with higher abundance were detected in cloacal samples, such as *Fur*, O-linked flagellar glycosylation, *CheA / CheY,* Siderophore-dependent iron uptake system, Ton system, and *VctPDGC* system. All these virulence genes encoding proteins were related to flagellar, iron uptake, and transport. Flagella are typically regarded as significant virulence factors that can facilitate motility and chemotaxis, allowing bacteria to travel to more favorable environments (Duan et al., 2013; Nakamura and Minamino, 2019). Iron is an important element for survival and colonization by bacteria since it plays a crucial role in the electron transport chain to produce energy (Andrews et al., 2003). Iron acquisition systems are used by bacteria to scavenge iron from the environment under iron-restricted conditions (Andrews et al., 2003; Krewulak and Vogel, 2008). Therefore, successful competition for iron is crucial for pathogenicity. These virulence factors may be important potential factors causing diseases in sea turtles.

This study also has its shortcomings. Although we collected three sea turtles for comparative analysis, due to their different distributions in the study area, the numbers of each type of sea turtle were uneven, which might be the reason for the differences in the bacterial communities of sea turtles. In addition, we have detected pathogenic bacteria in sea turtles, but have not yet successfully isolated potential pathogenic bacteria. This can serve as one of the directions for further research in the future.

## Conclusion

Taken together, we first performed a detailed analysis of the oral, nasal, and cloacal microbiota composition and functional characteristics in three species of sea turtles, including green turtles, loggerheads, and hawksbills, using shotgun metagenomic sequencing. Our study suggested that the microbiota compositions and functions are significantly different among the three host species and tissue regions. It is worth noting that we have identified some potential pathogenic bacteria, detected several ARGs and virulence genes associated with pathogenicity. These may pose potential risks to the health of sea turtles and could also be transmitted to the marine environment and even humans through sea turtles as vectors, which deserves our attention. The findings in our study demonstrate that the microbial composition and function in these sea turtles exhibit both species-specific and region-specific variations. The implications and underlying mechanisms of these associations offer valuable insights for future research on the sea turtle microbiome.

## Data Availability

The raw metagenomic datasets in this study are publicly available in the NCBI BioProject (https://www.ncbi.nlm.nih.gov/bioproject) with the accession PRJNA1232438.

## Footnotes

^1^ https://www.iucnredlist.org

^2^ https://github.com/OpenGene/fastp

^3^ http://bowtie-bio.sourceforge.net/bowtie2/index.shtml

^4^ http://topaz.gatech.edu/GeneMark/

^5^ https://github.com/bbuchfink/diamond/

^6^ https://www.ncbi.nlm.nih.gov/

^7^ http://www.kegg.jp/kegg/

^8^ http://eggnogdb.embl.de/#/app/home

^9^ http://www.cazy.org/

^10^ http://www.mgc.ac.cn/VFs/main.htm

^11^ https://card.mcmaster.ca/

^12^ http://bacdive.dsmz.de

## Supplementary material

FIGURE S1 Overview of commonalities and differences among all samples. **(A,B)** Core-pan gene rarefaction curve. The curve nearly plateaued when sufficient sequence data were input. **(C)** Heatmap showing the correlation between the samples and the gene table. **(D)** The gene numbers among different groups. We divided all samples into six groups, HG. G represents cloacal samples of green turtles, HG. B represents nasal samples of green turtles, HG. K represents oral samples of green turtles, DM. G, DM. B, DM. K represent cloacal, nasal, and oral samples of hawksbills, XG. G, XG. B, XG. K represent cloacal, nasal, oral samples of loggerheads.

FIGURE S2 The top 30 bacteria from phylum to species level across all samples. From **(A–F)** are from the phylum to the species level.

FIGURE S3 The alpha diversity analysis of the sea turtle bacterial community from the class to species levels based on turtle species and tissue regions. **(A,B)** The Chao 1 index and Shannon diversity index across three turtle species. **(C,D)** The Chao 1 index and Shannon diversity index across three tissue regions. *p*-values indicate the confidence level of statistical analyses, with *p* < 0.05 indicating statistically significant differences. ns represents no significant differences, * represents *p* < 0.05, ** represents *p* < 0.01, and *** represents *p* < 0.001.

FIGURE S4 The beta diversity analysis of the sea turtle bacterial community from the class to species levels based on turtle species and tissue regions. **(A,B)** PCoA results based on three species and tissue regions, respectively. **(C,D)** ANOSIM results based on three species and tissue regions, respectively.

FIGURE S5 The beta diversity analysis of the sea turtle bacterial community from phylum to species levels **(A–F)** in the same tissue area based on different sea turtles. HG. G represents cloacal samples of green turtles, HG. B represents nasal samples of green turtles, HG. K represents oral samples of green turtles, DM. G, DM. B, DM. K represent cloacal, nasal, and oral samples of hawksbills, XG. G, XG. B, XG. K represent cloacal, nasal, oral samples of loggerheads.

FIGURE S6 **(A,B)** The relative abundance of the top 10 bacterial phyla and top 30 classes, orders, families, genera, and species based on three turtle species and tissue regions. **(C,D)** The shared and unique bacterial taxa from phylum to species based on three turtle species and tissue regions.

FIGURE S7 The overview functional characteristics of the sea turtle microbiome. **(A)** The gene counts annotated in KEGG, eggNOG, CAZY, CARD, and VFDB databases. **(B)** The KEGG pathway annotation results. **(C)** The COG function annotation results. **(D)** The CAZY family annotation results. **(E)** The ARO resistance mechanism prediction results. **(F)** The virulence factors prediction results

FIGURE S8 **(A,B)** The abundance of the top 20 KEGG pathways based on three turtle species and sampling locations. **(C,D)** The abundance of the CAZY families based on three turtle species and tissue regions. **(E,F)** The abundance of the top 20 ARGs based on three turtle species and tissue regions. **(G,H)** The abundance of the top 20 virulence factors based on three turtle species and tissue regions. **(I,J)** The shared and unique ARGs based on three turtle species and tissue regions. **(K,L)** The shared and unique virulence factors based on three turtle species and tissue regions.
