# Correction: Metagenomic insights reveal the differences in the community composition and functional characteristics of the sea turtle microbiomes based on host species and tissue region

**DOI:** 10.3389/fmicb.2025.1721706

**Published:** 2025-10-21

**Authors:** Frontiers Production Office

**Affiliations:** Frontiers Media SA, Lausanne, Switzerland

**Keywords:** sea turtles, *Chelonia mydas*, *Caretta caretta*, *Eretmochelys imbricata*, metagenomic microbiome

Reviewer Vanessa Maria Bachmann was erroneously listed as being affiliated with the University of Haifa, Israel. The correct affiliation is University of Pavia, Italy.

The original version of this article has been updated.

