# Supplementary figures and images for: Metagenomic insights reveal the differences in the community composition and functional characteristics of the sea turtle microbiomes based on host species and tissue region
Source: Front Microbiol. 2025 Oct 3;16:1652229. doi: 10.3389/fmicb.2025.1652229 (PMC12531146; doi:10.3389/fmicb.2025.1652229)

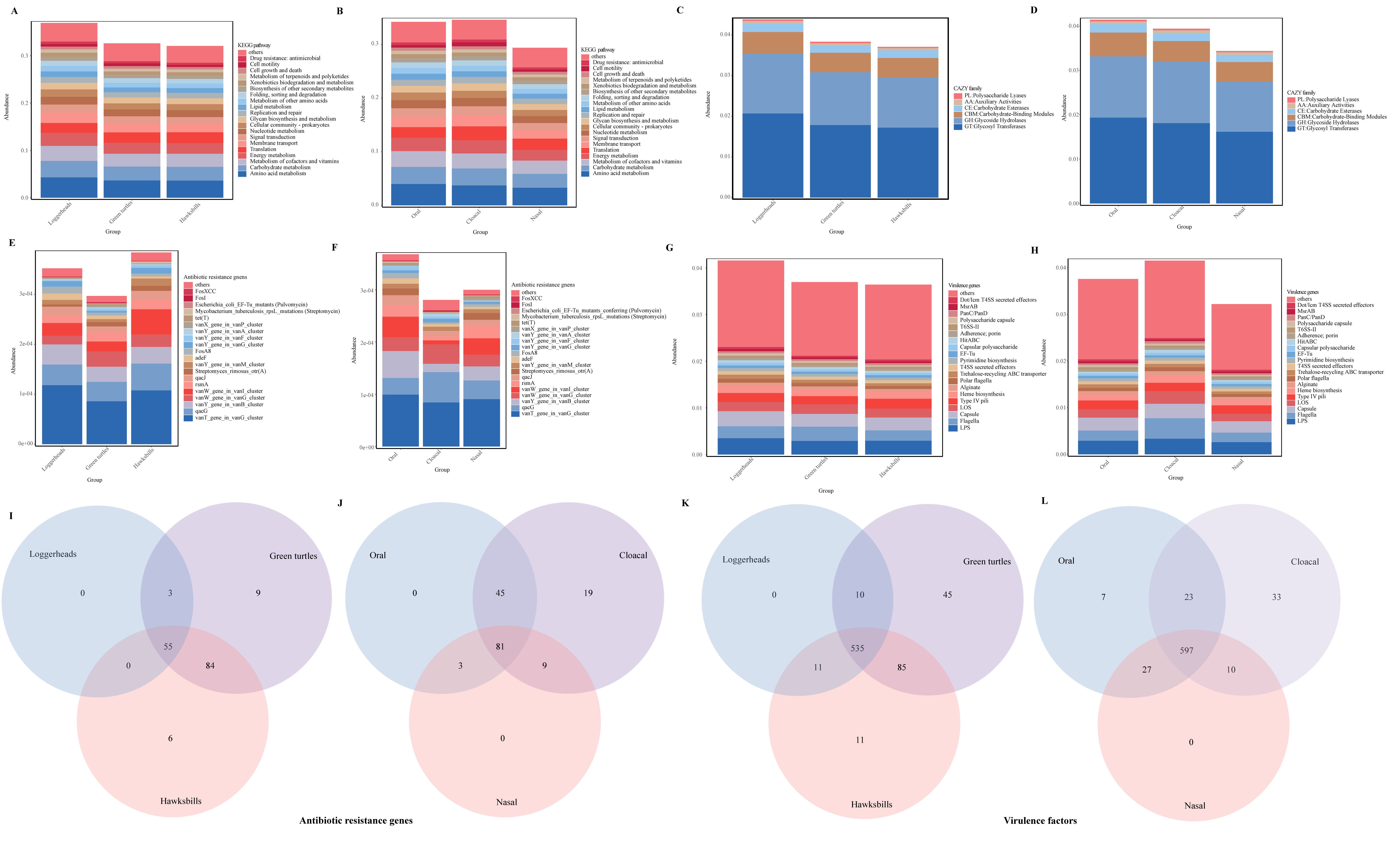

Supplement: FIGURE S8 — (A,B) The abundance of the top 20 KEGG pathways based on three turtle species and sampling locations. (C,D) The abundance of the CAZY families based on three turtle species and tissue regions. (E,F) The abundance of the top 20 ARGs based on three turtle species and tissue regions. (G,H) The abundance of the top 20 virulence factors based on three turtle species and tissue regions. (I,J) The shared and unique ARGs based on three turtle species and tissue regions. (K,L) The shared and unique virulence factors based on three turtle species and tissue regions. [file Data_Sheet_1.zip › Supplemental figures/Figure S8.tif]

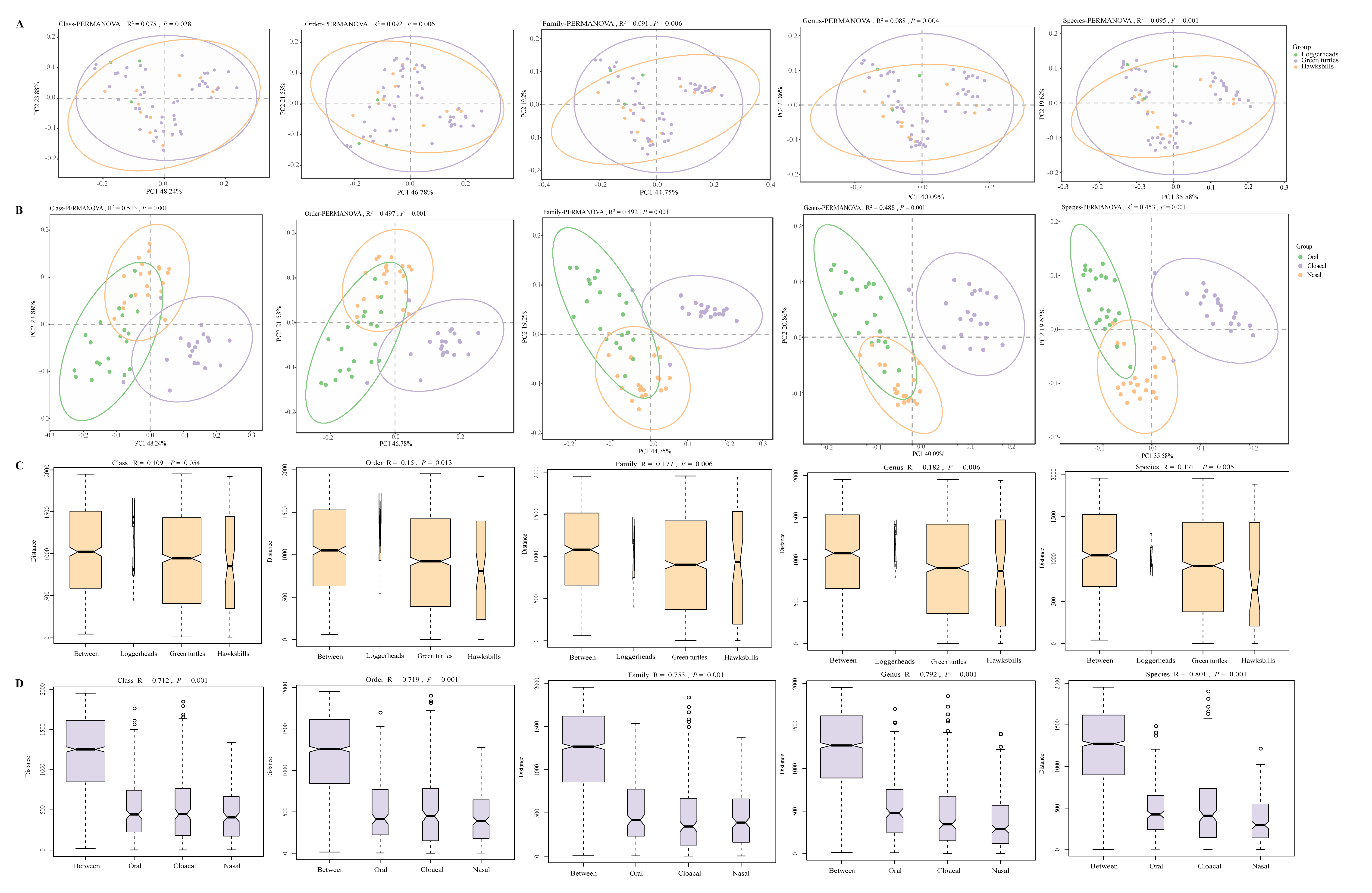

Supplement: FIGURE S8 — (A,B) The abundance of the top 20 KEGG pathways based on three turtle species and sampling locations. (C,D) The abundance of the CAZY families based on three turtle species and tissue regions. (E,F) The abundance of the top 20 ARGs based on three turtle species and tissue regions. (G,H) The abundance of the top 20 virulence factors based on three turtle species and tissue regions. (I,J) The shared and unique ARGs based on three turtle species and tissue regions. (K,L) The shared and unique virulence factors based on three turtle species and tissue regions. [file Data_Sheet_1.zip › Supplemental figures/Figure S4.tif]

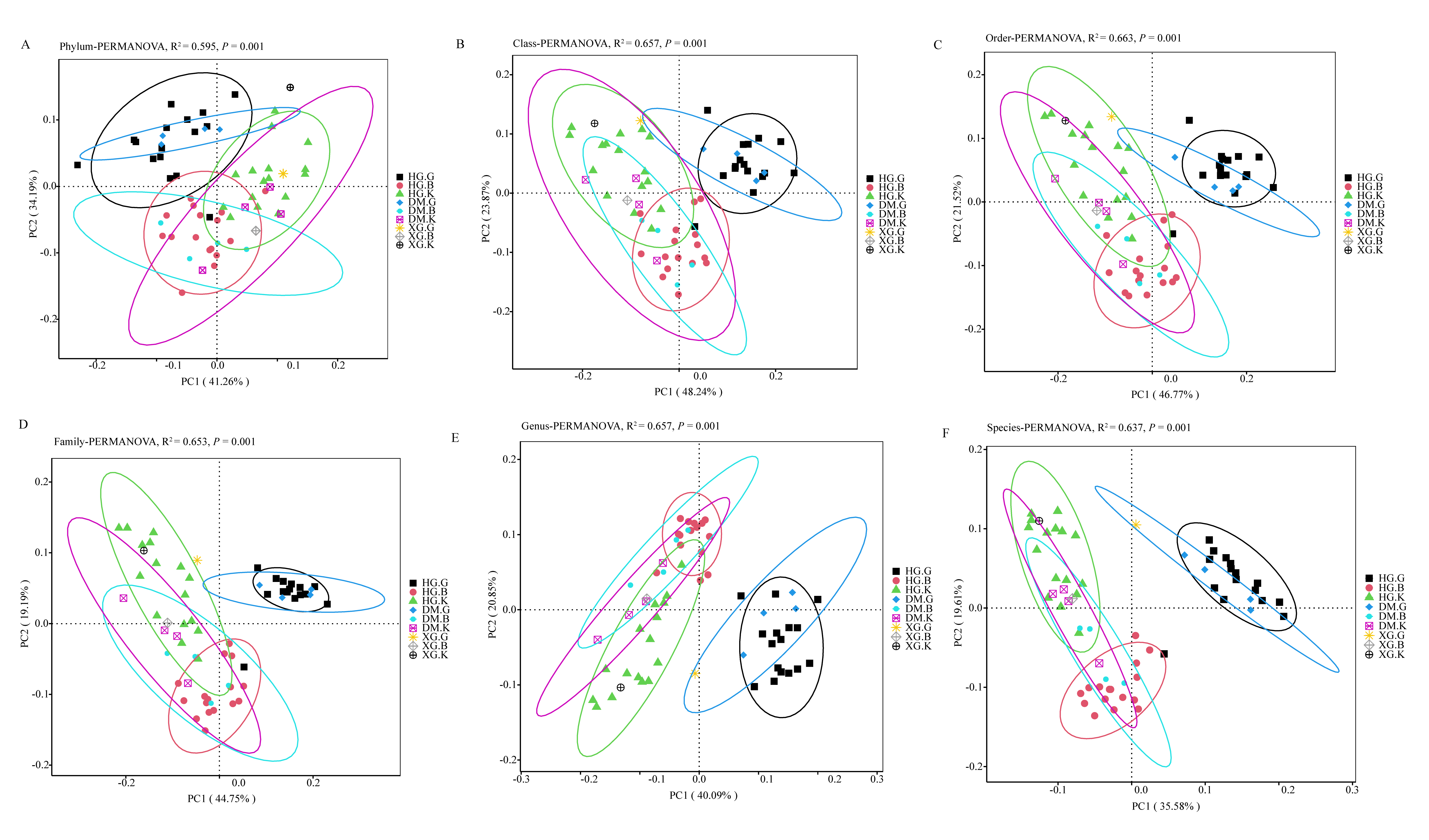

Supplement: FIGURE S8 — (A,B) The abundance of the top 20 KEGG pathways based on three turtle species and sampling locations. (C,D) The abundance of the CAZY families based on three turtle species and tissue regions. (E,F) The abundance of the top 20 ARGs based on three turtle species and tissue regions. (G,H) The abundance of the top 20 virulence factors based on three turtle species and tissue regions. (I,J) The shared and unique ARGs based on three turtle species and tissue regions. (K,L) The shared and unique virulence factors based on three turtle species and tissue regions. [file Data_Sheet_1.zip › Supplemental figures/Figure S5.tif]

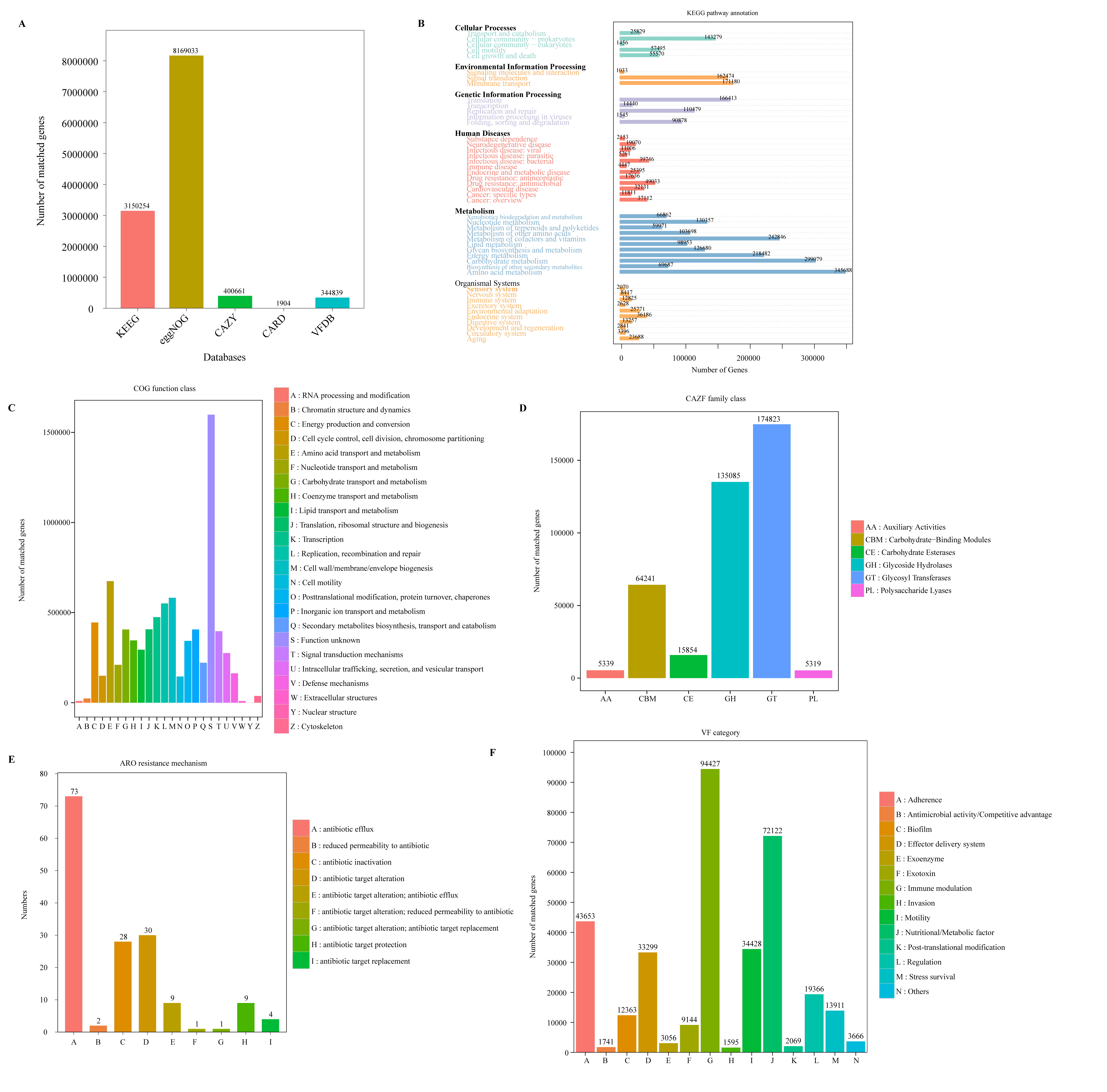

Supplement: FIGURE S8 — (A,B) The abundance of the top 20 KEGG pathways based on three turtle species and sampling locations. (C,D) The abundance of the CAZY families based on three turtle species and tissue regions. (E,F) The abundance of the top 20 ARGs based on three turtle species and tissue regions. (G,H) The abundance of the top 20 virulence factors based on three turtle species and tissue regions. (I,J) The shared and unique ARGs based on three turtle species and tissue regions. (K,L) The shared and unique virulence factors based on three turtle species and tissue regions. [file Data_Sheet_1.zip › Supplemental figures/Figure S7.tif]

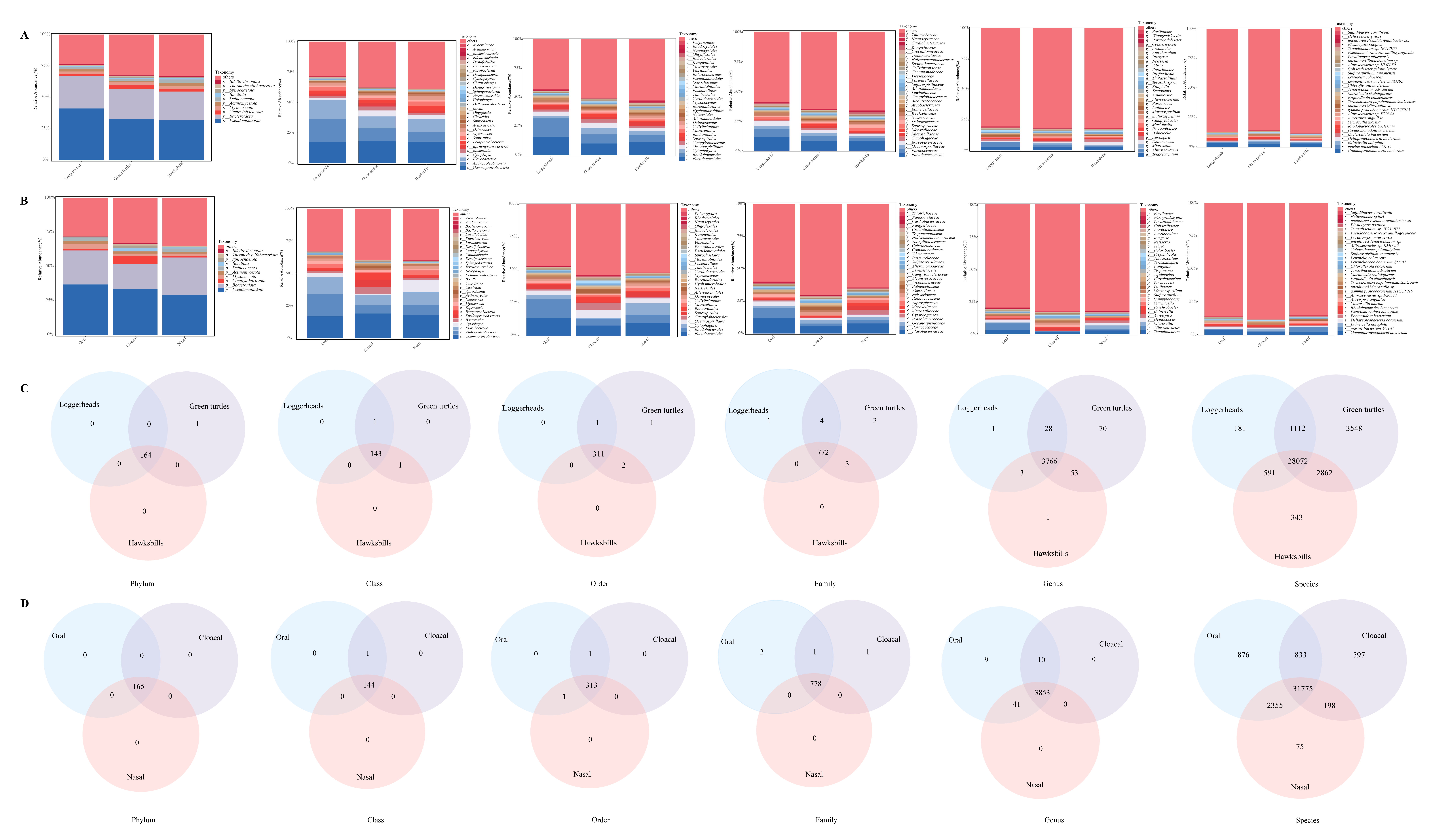

Supplement: FIGURE S8 — (A,B) The abundance of the top 20 KEGG pathways based on three turtle species and sampling locations. (C,D) The abundance of the CAZY families based on three turtle species and tissue regions. (E,F) The abundance of the top 20 ARGs based on three turtle species and tissue regions. (G,H) The abundance of the top 20 virulence factors based on three turtle species and tissue regions. (I,J) The shared and unique ARGs based on three turtle species and tissue regions. (K,L) The shared and unique virulence factors based on three turtle species and tissue regions. [file Data_Sheet_1.zip › Supplemental figures/Figure S6.tif]

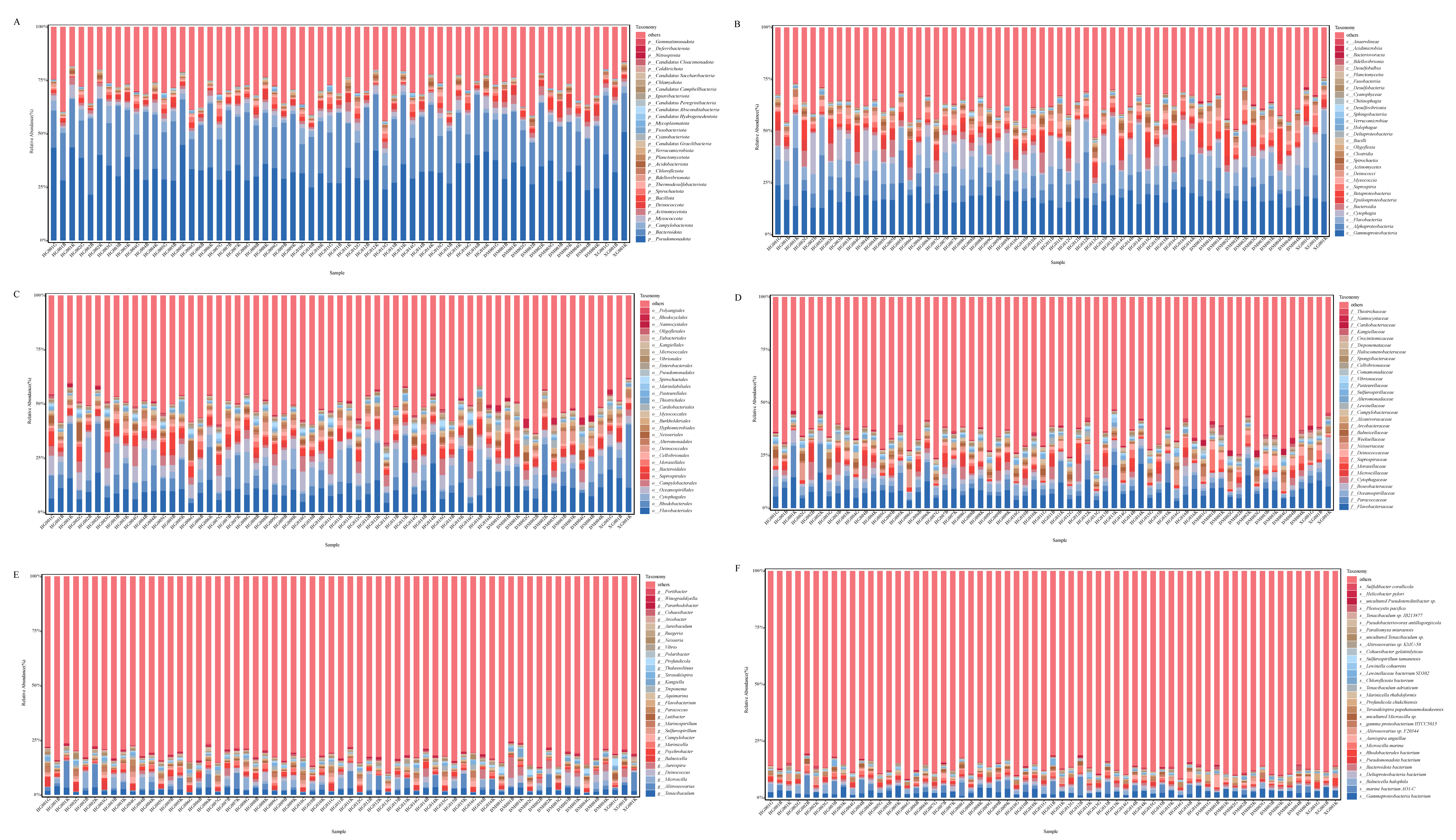

Supplement: FIGURE S8 — (A,B) The abundance of the top 20 KEGG pathways based on three turtle species and sampling locations. (C,D) The abundance of the CAZY families based on three turtle species and tissue regions. (E,F) The abundance of the top 20 ARGs based on three turtle species and tissue regions. (G,H) The abundance of the top 20 virulence factors based on three turtle species and tissue regions. (I,J) The shared and unique ARGs based on three turtle species and tissue regions. (K,L) The shared and unique virulence factors based on three turtle species and tissue regions. [file Data_Sheet_1.zip › Supplemental figures/Figure S2.tif]

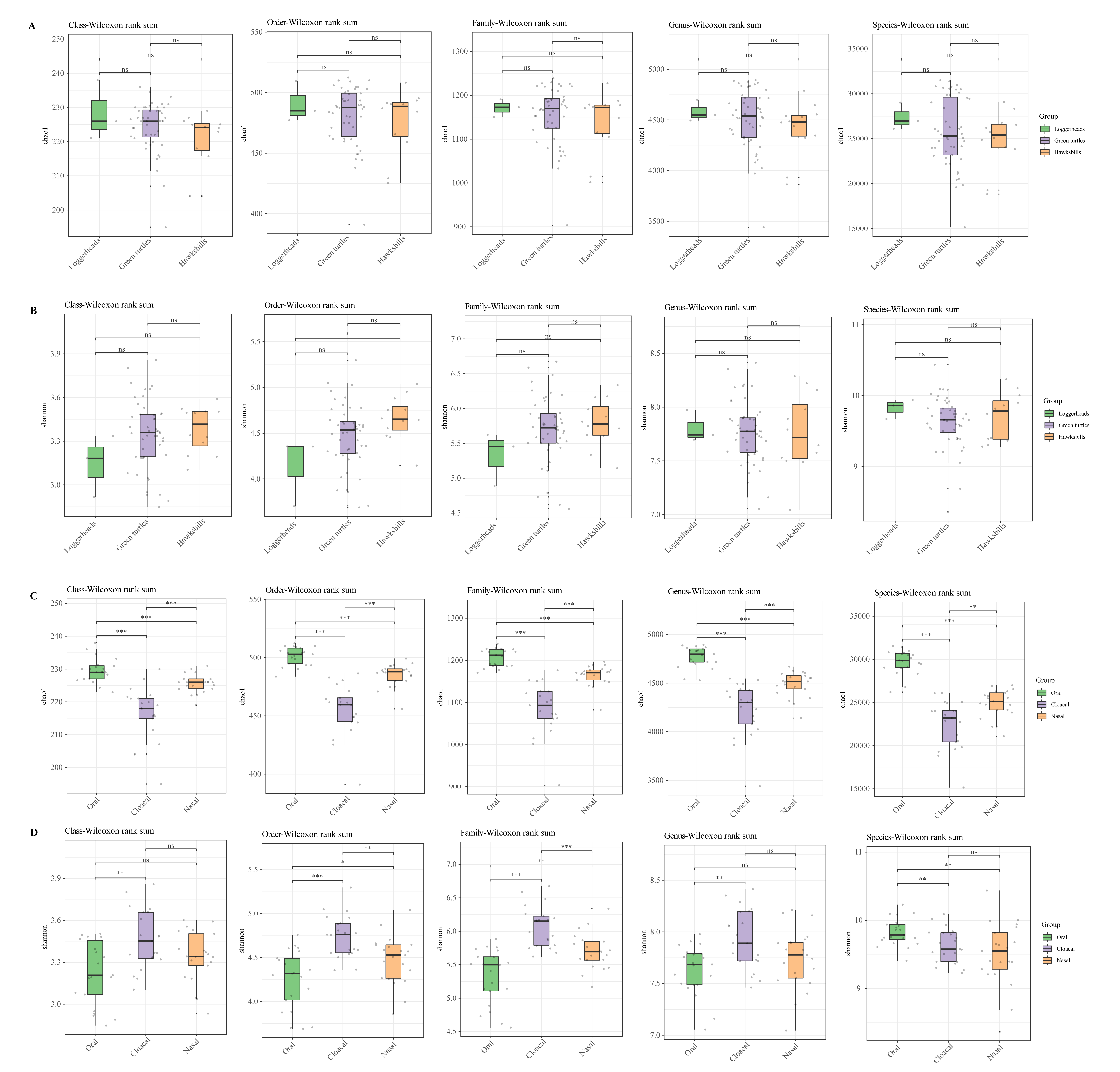

Supplement: FIGURE S8 — (A,B) The abundance of the top 20 KEGG pathways based on three turtle species and sampling locations. (C,D) The abundance of the CAZY families based on three turtle species and tissue regions. (E,F) The abundance of the top 20 ARGs based on three turtle species and tissue regions. (G,H) The abundance of the top 20 virulence factors based on three turtle species and tissue regions. (I,J) The shared and unique ARGs based on three turtle species and tissue regions. (K,L) The shared and unique virulence factors based on three turtle species and tissue regions. [file Data_Sheet_1.zip › Supplemental figures/Figure S3.tif]

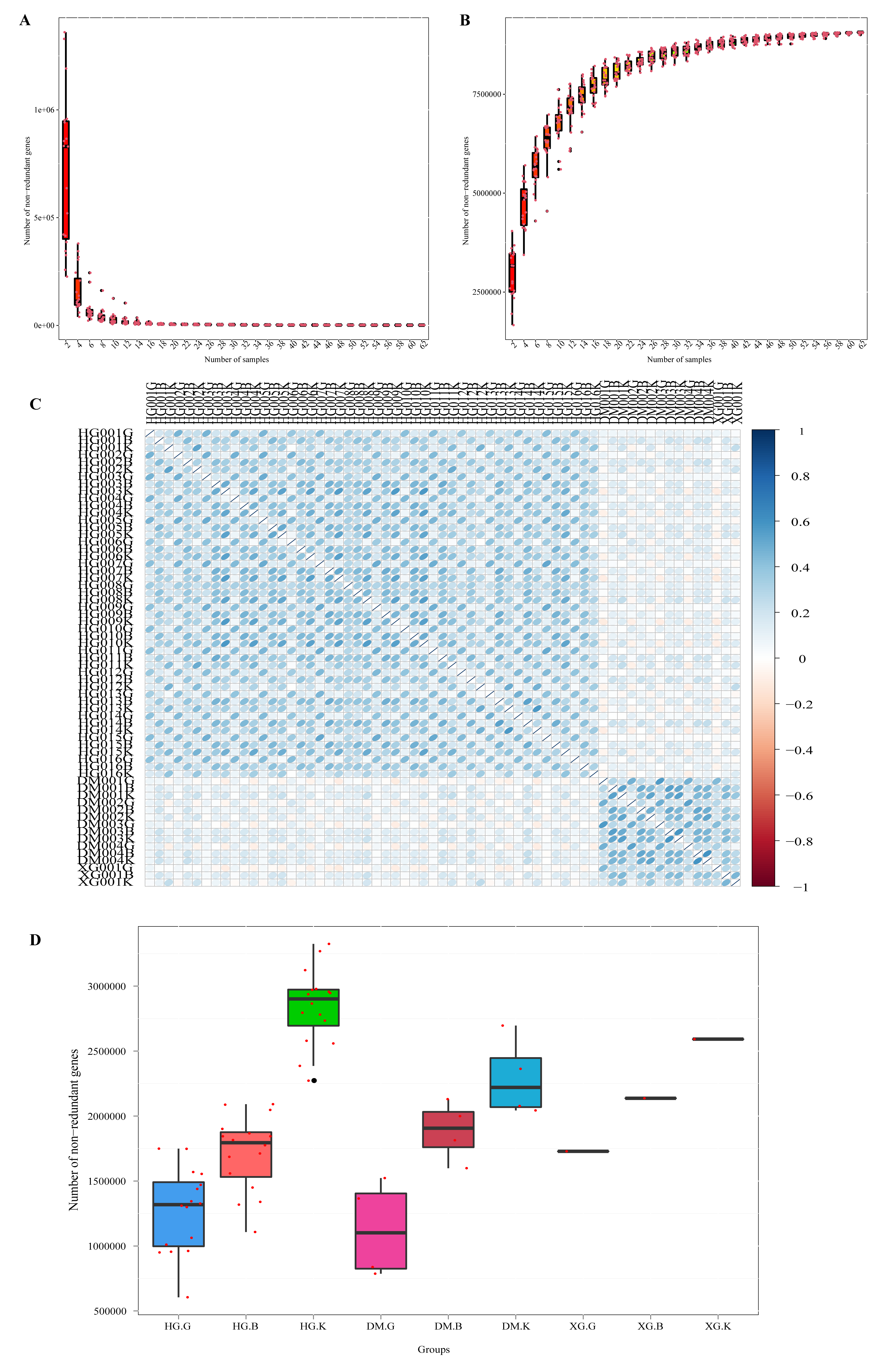

Supplement: FIGURE S8 — (A,B) The abundance of the top 20 KEGG pathways based on three turtle species and sampling locations. (C,D) The abundance of the CAZY families based on three turtle species and tissue regions. (E,F) The abundance of the top 20 ARGs based on three turtle species and tissue regions. (G,H) The abundance of the top 20 virulence factors based on three turtle species and tissue regions. (I,J) The shared and unique ARGs based on three turtle species and tissue regions. (K,L) The shared and unique virulence factors based on three turtle species and tissue regions. [file Data_Sheet_1.zip › Supplemental figures/Figure S1.tif]
